# Supplementary material for: Is type of work associated with physical activity and sedentary behaviour in women with fibromyalgia? A cross-sectional study from the al-Ándalus project
Source: BMJ Open. 2020 Apr 28;10(4):e034697. doi: 10.1136/bmjopen-2019-034697 (PMC7213859; doi:10.1136/bmjopen-2019-034697)
Supplement: Supplementary data [file bmjopen-2019-034697supp001.pdf]

STROBE Statement—checklist of items that should be included in reports of observational studies

	Item No.	Recommendation	Page No.	Relevant text from manuscript
**Title and abstract**	1	(*a*) Indicate the study's design with a commonly used term in the title or the abstract	1	*"This cross-sectional study involved 258 women with fibromyalgia…"*
		(*b*) Provide in the abstract an informative and balanced summary of what was done and what was found	1	Methods and results sections from the abstract
**Introduction**				
Background/rationale	2	Explain the scientific background and rationale for the investigation being reported	2-3	Mainly 2nd and 3rd paragraph in the introduction section
Objectives	3	State specific objectives, including any prespecified hypotheses	3	*"Therefore, the aim of the present study was to analyze the association between the type of work (productive vs. reproductive), physical activity intensity levels (in leisure time, home, work, and total), and sedentary behavior in women with fibromyalgia"*
**Methods**				
Study design	4	Present key elements of study design early in the paper	3-4	1st and 2nd paragraph in the methods section
Setting	5	Describe the setting, locations, and relevant dates, including periods of recruitment, exposure, follow-up, and data collection	3-4	1st and 2nd paragraph in the methods section
Participants	6	(*a*) *Cohort study*—Give the eligibility criteria, and the sources and methods of selection of participants. Describe methods of follow-up<br>*Case-control study*—Give the eligibility criteria, and the sources and methods of case ascertainment and control selection. Give the rationale for the choice of cases and controls<br>*Cross-sectional study*—Give the eligibility criteria, and the sources and methods of selection of participants	3	*"The inclusion criteria for the present study were: i) to have been diagnosed with fibromyalgia; ii) to meet the 1990 ACR criteria [10]; iii) not to suffer from any acute or terminal illness, or serious cognitive impairment; iv) to be a woman."*
		(*b*) *Cohort study*—For matched studies, give matching criteria and number of exposed and unexposed<br>*Case-control study*—For matched studies, give matching criteria and the number of controls per case	N/A	

Girela-Rejón MJ, *et al. BMJ Open* 2020; 10:e034697. doi: 10.1136/bmjopen-2019-034697

Variables	7	Clearly define all outcomes, exposures, predictors, potential confounders, and effect modifiers. Give diagnostic criteria, if applicable	4-7	Each paragraph describes diagnostic criteria, outcomes, predictors, and potential confounders.
Data sources/ measurement	8*	For each variable of interest, give sources of data and details of methods of assessment (measurement). Describe comparability of assessment methods if there is more than one group	4-7	Each paragraph describes assessment method of diagnostic criteria, outcomes, predictors, and potential confounders.
Bias	9	Describe any efforts to address potential sources of bias		
Study size	10	Explain how the study size was arrived at	3 and 8	Initial sample size in page 3 *"This study belongs to al-Ándalus project, in which a representative sample of women with fibromyalgia from Andalusia (Spain) was examined [17]."* Final sample size in page 8 "*The flowchart of the participants included in the present study is shown in Figure 1."*

Quantitative variables	11	Explain how quantitative variables were handled in the analyses. If applicable, describe which groupings were chosen and why	7	Two paragraphs included in the statistical analysis section
Statistical methods	12	(*a*) Describe all statistical methods, including those used to control for confounding	7	Two paragraphs included in the statistical analysis section
		(*b*) Describe any methods used to examine subgroups and interactions	N/A	
		(*c*) Explain how missing data were addressed	Figure 1	
		(*d*) *Cohort study*—If applicable, explain how loss to follow-up was addressed<br>*Case-control study*—If applicable, explain how matching of cases and controls was addressed<br>*Cross-sectional study*—If applicable, describe analytical methods taking account of sampling strategy	3	*"This study belongs to al-Ándalus project, in which a representative sample of women with fibromyalgia from Andalusia (Spain) was examined [17]."*
		(*e*) Describe any sensitivity analyses	8	*"age, fat percentage, education level, and marital status were included as covariates in all the analyses described below. Unadjusted analyses and analyses additionally*

Girela-Rejón MJ, *et al. BMJ Open* 2020; 10:e034697. doi: 10.1136/bmjopen-2019-034697

				accounting for disease severity are included as supplementary material"
**Results**				
Participants	13*	(a) Report numbers of individuals at each stage of study—eg numbers potentially eligible, examined for eligibility, confirmed eligible, included in the study, completing follow-up, and analysed	8	*"The flowchart of the participants included in the present study is shown in Figure 1."*
		(b) Give reasons for non-participation at each stage	8	*"The flowchart of the participants included in the present study is shown in Figure 1."*
		(c) Consider use of a flow diagram	8	*"The flowchart of the participants included in the present study is shown in Figure 1."*
Descriptive data	14*	(a) Give characteristics of study participants (eg demographic, clinical, social) and information on exposures and potential confounders	20	Table 1
		(b) Indicate number of participants with missing data for each variable of interest	8	*"The flowchart of the participants included in the present study is shown in Figure 1."*
		(c) *Cohort study*—Summarise follow-up time (eg, average and total amount)	N/A	
Outcome data	15*	*Cohort study*—Report numbers of outcome events or summary measures over time	N/A	
		Case-control study—Report numbers in each exposure category, or summary measures of exposure	N/A	
		Cross-sectional study—Report numbers of outcome events or summary measures	20	Table 1
Main results	16	(*a*) Give unadjusted estimates and, if applicable, confounder-adjusted estimates and their precision (eg, 95% confidence interval). Make clear which confounders were adjusted for and why they were included	7	Supplementary table 2 and page 7 *"Given that significant differences between groups emerged for all these variables (with the exception of total number of tender points), age, fat percentage, education level, and marital status were included as covariates in all the analyses described below."*
		(*b*) Report category boundaries when continuous variables were categorized	N/A	
		(*c*) If relevant, consider translating estimates of relative risk into absolute risk for a meaningful time period	N/A	
Other analyses	17	Report other analyses done—eg analyses of subgroups and interactions, and sensitivity analyses	7	*"Unadjusted analyses and analyses additionally accounting for disease severity*

				are included as supplementary material (supplementary tables 2 and 3)".
**Discussion**				
Key results	18	Summarise key results with reference to study objectives	9	1st paragraph in the discussion section
Limitations	19	Discuss limitations of the study, taking into account sources of potential bias or imprecision. Discuss both direction and magnitude of any potential bias	12	Limitations paragraph "*The conclusions of the present study must be taken bearing its limitations in mind. A cross-sectional design was used, so the results cannot be deemed as causal….*"
Interpretation	20	Give a cautious overall interpretation of results considering objectives, limitations, multiplicity of analyses, results from similar studies, and other relevant evidence	13	Last paragraph of the discussion *"Given the cross-sectional design of this study, future longitudinal research is warranted in order to address the causality of the present findings. If they are corroborated…"*
Generalisability	21	Discuss the generalisability (external validity) of the study results	13	Last paragraph of the discussion
**Other information**				
Funding	22	Give the source of funding and the role of the funders for the present study and, if applicable, for the original study on which the present article is based	13	Funding paragraph *"This work was supported by the Spanish Ministries of Economy and Competitiveness (I+D+i DEP2010-15639; I+D+i DEP2013-40908-R) …. The funders of this study did not have any role in the study design, data collection and analyses, decision to publish or preparation of the manuscript."*

*Give information separately for cases and controls in case-control studies and, if applicable, for exposed and unexposed groups in cohort and cross-sectional studies.

Note: An Explanation and Elaboration article discusses each checklist item and gives methodological background and published examples of transparent reporting. The STROBE checklist is best used in conjunction with this article (freely available on the Web sites of PLoS Medicine at http://www.plosmedicine.org/, Annals of Internal Medicine at http://www.annals.org/, and Epidemiology at http://www.epidem.com/). Information on the STROBE Initiative is available at www.strobe-statement.org.

Girela-Rejón MJ, *et al. BMJ Open* 2020; 10:e034697. doi: 10.1136/bmjopen-2019-034697
