# Supplementary material for: Is type of work associated with physical activity and sedentary behaviour in women with fibromyalgia? A cross-sectional study from the al-Ándalus project
Source: BMJ Open. 2020 Apr 28;10(4):e034697. doi: 10.1136/bmjopen-2019-034697 (PMC7213859; doi:10.1136/bmjopen-2019-034697)
Supplement: Supplementary data [file bmjopen-2019-034697supp002.pdf]

Supplementary Table 2. Mean difference in physical activity variables between women with fibromyalgia with a reproductive work only (*n*=143) and those with a reproductive + productive work (*n*=115)

	Light Intensity						Moderate Intensity						Vigorous Intensity					
	Mean difference	95 % CI		P_1	P_2	P_3	Mean difference	95 % CI		P_1	P_2	P_3	Mean difference	95 % CI		P_1	P_2	P_3
Leisure-time PA	4.875	-30.458	40.208	0.786	0.889	0.513	-10.967	-35.706	13.771	0.383	0.355	0.558	-5.980	-16.720	4.760	0.274	0.221	0.268
PA at home	**491.941**	**307.317**	**676.565**	**<0.001**	**<0.001**	**<0.001**	**165.643**	**38.513**	**292.773**	**0.011**	0.081	0.088	6.539	-55.049	68.127	0.835	0.751	0.787
PA at work	**-256.389**	**-502.304**	**-10.473**	**0.041**	**0.001**	**0.002**	**154.676**	**19.318**	**290.033**	**0.025**	0.142	0.135	0.559	-63.411	64.529	0.986	0.920	0.940
Total PA	**-752.616**	**-1000.376**	**-504.857**	**<0.001**	**<0.001**	**<0.001**	**-261.011**	**-445.530**	**-76.492**	**0.006**	**0.004**	**0.006**	-79.799	-166.660	7.061	0.072	0.089	0.079

CI: confidence interval. PA: physical activity. Reproductive + productive work was used as reference category in all comparisons.

Mean difference and P_1 show the results for unadjusted analysis

P_2: Analysis adjusted for age, fat percentage, education level, and marital status

P_3: Analysis adjusted for age, fat percentage, education level, marital status, and disease severity

Supplementary Table 3. Mean difference in sedentary behaviour variables between women with fibromyalgia with a reproductive work only (*n*=143) and those with a reproductive + productive work (*n*=115)

	Mean difference	95 % CI		P_1	P_2	P_3
Total sedentary Time (Monday to Friday)	-312.514	-632.988	7.959	0.056	0.308	0.207
Total sedentary Time (Weekend)	-63.736	-190.825	63.353	0.324	0.749	0.880

CI: confidence interval. Reproductive + productive work was used as reference category in all comparisons.

Mean difference and P_1 show the results for unadjusted analysis

P_2: Analysis adjusted for age, fat percentage, education level, and marital status

P_3: Analysis adjusted for age, fat percentage, education level, marital status, and total disease severity
