# Supplementary material for: Is type of work associated with physical activity and sedentary behaviour in women with fibromyalgia? A cross-sectional study from the al-Ándalus project
Source: BMJ Open. 2020 Apr 28;10(4):e034697. doi: 10.1136/bmjopen-2019-034697 (PMC7213859; doi:10.1136/bmjopen-2019-034697)
Supplement: Author's manuscript [file bmjopen-2019-034697.draft_revisions.pdf]

BMJ Open

BMJ Open is committed to open peer review. As part of this commitment we make the peer review history of every article we publish publicly available.

When an article is published we post the peer reviewers' comments and the authors' responses online. We also post the versions of the paper that were used during peer review. These are the versions that the peer review comments apply to.

The versions of the paper that follow are the versions that were submitted during the peer review process. They are not the versions of record or the final published versions. They should not be cited or distributed as the published version of this manuscript.

BMJ Open is an open access journal and the full, final, typeset and author-corrected version of record of the manuscript is available on our site with no access controls, subscription charges or pay-per-view fees (http://bmjopen.bmj.com).

If you have any questions on BMJ Open's open peer review process please email info.bmjopen@bmj.com

BMJ Open

Is type of work associated with physical activity and sedentary behavior in women with fibromyalgia? A cross-sectional study from the al-Ándalus project.

Journal:	*BMJ Open*
Manuscript ID	bmjopen-2019-034697
Article Type:	Original research
Date Submitted by the Author:	02-Oct-2019
Complete List of Authors:	Girela-Rejón, Maria José; University of Granada, Department of Didactic of Corporal Expression, Faculty of Education Sciences<br>Gavilán Carrera, Blanca; University of Granada, Department of Physical Education and Sport, Faculty of Sport Sciences; Sport and Health University Research Institute (iMUDS)<br>Aparicio-Ortega, Esther; University of Granada, Department of Physical Education and Sport, Faculty of Sport Sciences<br>Borges-Cosic, Milkana; University of Granada, Department of Physical Education and Sport, Faculty of Sport Sciences; Sport and Health University Research Institute (iMUDS)<br>García-Rodríguez, Inmaculada C; University of Granada, Department of Physical Education and Sport, Faculty of Sport Sciences<br>Delgado-Fernández, Manuel; University of Granada, Department of Physical Education and Sport, Faculty of Sport Sciences; Sport and Health University Research Institute (iMUDS)<br>Estévez-López, Fernando; Erasmus Medical Center, Department of Child and Adolescent Psychiatry/Psychology
Keywords:	RHEUMATOLOGY, Pain management < ANAESTHETICS, SPORTS MEDICINE

SCHOLARONE™
Manuscripts

Title: Is type of work associated with physical activity and sedentary behavior in women with fibromyalgia? A cross-sectional study from the al-Ándalus project.

Authors:

Maria José Girela-Rejón, PhD[1*], Blanca Gavilán-Carrera, MSc[2,3*], Esther Aparicio-Ortega, MSc[2], Milkana Borges-Cosic, PhD[2,3], Inmaculada C García-Rodríguez, MSc[2,3], Manuel Delgado-Fernández, PhD[2,3], Fernando Estévez-López, PhD[4].

Affiliations:

1 Department of Didactic of Corporal Expression, Faculty of Education Sciences, University of Granada, Spain.

2 Department of Physical Education and Sport, Faculty of Sport Sciences, University of Granada, Granada, Spain.

3 Sport and Health University Research Institute (iMUDS)

4 Department of Child and Adolescent Psychiatry/Psychology, Erasmus University Medical Center, Rotterdam, the Netherlands.

*These authors equally contributed to this work

Corresponding author

Fernando Estévez-López

Department of Child and Adolescent Psychiatry/Psychology, Erasmus University Medical Center, Rotterdam, the Netherlands

Email: festevez@ugr.es

Keywords: Chronic pain; Lifestyle; Rheumatic and musculoskeletal diseases; Women's health

For peer review only

Word count: 3625

ABSTRACT

Objectives: To analyze the association between the type of work (productive vs. reproductive work) and the levels of physical activity and sedentary behavior in women with fibromyalgia.

Method: This cross-sectional study involved 258 women with fibromyalgia from southern Spain. Of them, 55% performed reproductive work (unpaid, associated with care giving and domestic roles) exclusively, while 45% had a productive job (remunerated, that results in good or services). Physical activity of light, moderate and vigorous intensity in the leisure time, at home, at work, and total were measured through the Leisure Time Physical Activity Instrument (LTPAI) and with the Physical Activity at Home and Work Instrument (PAHWI), respectively. Sedentary behavior was measured by the Sedentary Behavior Questionnaire (SBQ).

Results: After adjusting for age, fat percentage, education level and marital status, the MANCOVA model informed the existence of significant differences between type of work groups (*P*<0.001). Women with a productive work engaged in more light physical activity at work (mean difference=448.52 min; 95 % CI 179.66 to 717.38; *P*=0.001), and total physical activity of light (809.72 min; 535.91 to 1085.53; *P*<0.001) and moderate (299.78; 97.31 to 502.25; *P*=0.004) intensity. Women with a reproductive work engaged in more light physical activity at home (379.14; 175.64 to 582.64; *P*<0.001). Leisure time physical activity and sedentary behavior was similar in both groups (*P*>0.05 for all comparisons).

Conclusions: Women with a productive work had greater levels of physical activity compared to those who only did reproductive work, except for physical activity at home. To have a productive work might facilitate that women with fibromyalgia move towards a more active lifestyle.

For peer review only

Strengths and limitations of this study

- The questionnaires used to assess physical activity are able to distinguish whether the behavior is being done at leisure time, home or work.
- A large sample of patients with fibromyalgia representative of southern Spain was included.
- The cross-sectional impedes determination of causal relationships.
- Self-administered questionnaires could provide misleading information in women with fibromyalgia.

INTRODUCTION

Reproductive work refers to all those tasks whose purpose is to take care and ensure the wellness of the family and household, reason why it is also called domestic work. Its main characteristics are: not to be remunerated by salary, to be mainly female work and to stay invisible [1]. Nevertheless, the economically and socially recognized work is the productive work (goods and services), which refers to all those human activities that have an exchange value and lead to financial remuneration [1,2].

The society in general transmits values and rules that shape female and male identities, so that work is differentiated by gender, men being more oriented to productive work and women being typically the main responsible for domestic work and family care, called reproductive work [3–5]. The majority of women imagine themselves performing activities associated with their gender and live with the aim of meeting the expectations related with their maternal role. Therefore, the major difference between genders has been and remains to be the reproductive work [6,7]. The lower visibility and social recognition of reproductive work also entails less economic independence as it is unpaid [1]. Despite women's increasing participation in the work market, this means to assume that women do the so-called double day, in which they face the difficulty of combining productive and reproductive work [3,8]

Fibromyalgia is a condition that affects 2.4% of the Spanish population, being more frequent among women (4.2%) than among men (0.25%) [9]. Fibromyalgia is characterized by widespread

For peer review only

musculoskeletal pain of unknown origin, as well as fatigue, joint stiffness and sleep problems, among others [10], what has negative consequences on their ability to work [11]. The idea that health problems are not randomly distributed, but influenced by gender inequality is becoming increasingly popular, and the social context of women who suffer from fibromyalgia is a society that assigns them the caring role (either for children or elderly). Thus, the family caring responsibilities and the double working day that some of these women perform (remunerated work and housework) are factors that also affect their health status [8]. For instance, those women who perform productive work experience better health than those who do not do that kind of work in terms of pain, fatigue, and depression [12]. Among the factors that could positively influence fibromyalgia symptoms, there is evidence that a higher level physical activity of different intensities and lower levels of sedentary behavior are associated with better symptomatology [13–15]. Unfortunately, the difficulties when reconciling productive and reproductive works could lead to a lack of time for those women [7], which is a potential barrier not to practice physical activity [16]. This potential relationship between work type and physical activity, which may determine health in this group of patients, is, however, not well-characterized yet.

Therefore, the aim of the present study was to analyze the association between the type of work (productive vs. reproductive), physical activity intensity levels (in leisure time, home, work, and total), and sedentary behavior in women with fibromyalgia.

METHOD

Design and participants

This study belongs to al-Ándalus project, in which a representative sample of women with fibromyalgia from Andalusia (Spain) was examined [17]. Associations from the eight Andalusian provinces were contacted and other participants were recruited through e-mail, letter, telephone or the university website. A total of 646 individuals with fibromyalgia were interested in participating in the study. The inclusion criteria for the present study were: i) to have been diagnosed with fibromyalgia; ii) to meet the 1990 ACR criteria [10]; iii) not to suffer from any acute or terminal illness, or serious

For peer review only

cognitive impairment; iv) to be a woman. The al-Ándalus project protocol was approved by the ethics committee of Virgen de las Nieves Hospital (Granada, Spain) with register number: 15/11/2013-N72. The guidelines of the Declaration of Helsinki (modified in 2000) were followed.

Procedure and instruments

The measurements were conducted in two non-consecutive days. On the first day, the participants signed an informed consent, completed a questionnaire on sociodemographic data, underwent the Mini-Mental State Examination (MMSE) [18] and were examined for tender points and body composition. Between days 1 and 2, they completed the following questionnaires at home: Leisure Time Physical Activity Instrument (LTPAI), Physical Activity at Home and Work Instrument (PAHWI) [19] and Sedentary Behavior Questionnaire (SBQ) [20]. On the second day, participants returned to the laboratory and questionnaires were checked by the research group.

Sociodemographic data

The sociodemographic data were obtained through a questionnaire that included items related to educational level, marital status and working status. Working status was classified as (i) patients with that only have a reproductive work (this is, unpaid and associated with care giving and domestic roles) or (ii) patients that also have a productive work (this is remunerated, that results in good or services). Women classified in productive work could do reproductive work at the same time.

Mini-Mental State Examination

It was used to detect severe cognitive impairment. Its score ranges between 0 and 30, and a score under 10 was considered as cognitive impairment. It is a short test for cognitive assessment that includes questions to evaluate five areas of the cognitive function: orientation, short-term memory, attention, concentration, long-term memory and language [18].

Tender points

For peer review only

The 18 tender points were assessed according to ACR guidelines [10] for the diagnosis of fibromyalgia using a standardized pressure algometer (FPK 20; Wagner Instruments, Greenwich, CT, USA). Increasing pressure was applied with the algometer on the selected area and the patients were requested to inform when they started to feel pain. The tender points were classified as positive when pain appeared at a pressure of $4kg/cm^2$ or lower. The same procedure was repeated twice and the mean was calculated. The total number of positive tender points was recorded for every patient. The participants needed to have a minimum of 11 positive points in order to confirm the diagnosis of fibromyalgia.

Fat percentage

The fat percentage was measured by means of a portable bioelectrical impedance analyzer with eight tactile electrodes (InBody R20, Biospace, Seoul, Korea). The participants were requested to refrain from taking a shower, performing intense physical activity or eating in the two hours prior to the measurement. They were asked to wear only underwear and no metal objects that could interfere in the measurement.

Leisure Time Physical Activity Instrument

The LTPAI is an instrument that has shown satisfactory reliability among patients with fibromyalgia [19]. It comprises four items with three intensity levels: light, moderate and vigorous. The participants were requested to indicate the number of hours a week they had performed leisure physical activity during the previous 4 weeks. The scale can be simplified in the following levels: i) 0.5 to 1.5 hours per week, ii) 2 to 4 hours per week, iii) > 4 hours per week. When a participant selected levels 1 or 2, the middle range value was used for the total score. When level 3 was selected, she was asked to provide the number of hours. When no level was selected, the number of hours was considered to be 0. The number of hours indicated by the participants for every intensity category was added up to obtain the leisure-time physical activity level during one week.

For peer review only

Physical Activity at Home and Work Instrument

The PAHWI questionnaire [19], comprises seven items with three categories for work performed at home (light, moderate and vigorous activity) and four categories for work performed out of home (sedentary, light, moderate and vigorous activity). The participants were asked to provide the average number of hours per week that they had spent on activities of each category in the previous four weeks. The hours of each category were added up to obtain the total score.

Total physical activity

Although the LTPAI and the PAHWI were administered separately, they both belong to a more complex questionnaire that covers all physical activity performed during the day: leisure time, housework, and work. The three categories (light, moderate and vigorous) of both questionnaires were summed up to obtain the total number of minutes of physical activity per week. The final result was divided by 7 to get the total number of minutes of physical activity per day.

Sedentary Behavior Questionnaire

The SBQ [20] informs about the time spent on 11 sedentary behaviors (watching television, sitting while eating, lying and resting, sitting while playing computer/video games, sitting while listening to music, sitting and talking on the phone, doing paperwork or office work, sitting and reading, playing a musical instrument, doing arts and crafts, sitting and driving/travelling in a car, bus, or train). The 11 items were completed separately for weekdays and weekend days. Response options were "none", "15 minutes or less", "30 minutes", "1 hour", "2 hours", "3 hours", "4 hours", "5 hours", or "6 hours or more". The time spent on each behavior was converted into hours (this is 15 minutes=0.25 hours). Hours per day for each item were summed separately for weekday and weekend days for the total scores of sedentary behaviors. For the summary variables of total hours/day spent in sedentary behaviors (weekday and weekend) and total sedentary hours/week, responses higher than 24 hours/day were truncated to 24 hours/ day.

For peer review only

Statistical analysis

Before main analyses and in order to identify potential confounders, the age, fat percentage, total number of tender points, education level (i.e., unfinished/primary studies or secondary/vocational/university studies) and marital status (i.e., currently married or unmarried) of participants with only reproductive work and those with a productive work were compared using unpaired samples t-test or χ^2 tests. Given that significant differences between groups emerged for all these variables (with the exception of total number of tender points), age, fat percentage, education level, and marital status were included as covariates in all the analyses described below.

A one-way multivariate analysis of covariance (MANCOVA) was conducted to compare the mean scores of women with fibromyalgia according to their type of work (i.e., participants with only reproductive work and those with a productive work) on (i) leisure time physical activity (of light, moderate, and vigorous physical activity), (ii) physical activity at home (of light, moderate, and vigorous physical activity), (iii) physical activity at work (i.e., the addition of housework and work) of light, moderate, and vigorous physical activity, (iv) total physical activity (i.e., the addition of leisure time, housework, and work) of light, moderate, and vigorous physical activity, (v) sedentary behavior during weekdays and (vi) sedentary behavior on the weekend. MANCOVA allows dependent variables to be correlated and is more powerful than ANCOVA for detecting group differences. Statistical significance was set at $P<0.05$ in all analyses. The Statistical Package for Social Sciences software (IBM SPSS, version 22.0) was used. Additionally, to illustrate our significant results, violin plots were created using R package ggplot2.

RESULTS

From the 646 potential participants from the initial sample, 39 were excluded for not having been diagnosed with fibromyalgia by a rheumatologist, 99 for not meeting the 1990 ACR criteria, 21 for being men, 1 due to severe cognitive impairment and 2 due to acute or terminal illness. From these 484 women, 36 had incomplete data related to fat percentage, physical activity or sedentary behavior.

For peer review only - http://bmjopen.bmj.com/site/about/guidelines.xhtml

Two were excluded for being students, 14 for being retired, 63 for being unable to work, 33 for being on sick leave and 78 for being unemployed. The final sample was composed of 258 women with fibromyalgia. Table 1 shows the sample characteristics. The majority of the participants were married (77.9%) and had low educational level (incomplete studies 9.7%; primary school 51.2%; secondary school 25.2%). 55.4% of the women performed reproductive work, while 44.6% performed productive work. The participants' mean age was 51 years old and their mean fat percentage was 40.2%. Significant differences emerged between groups (productive vs. reproductive) on age, body fat percentage, education level and marital status (all, P's ≤ 0.001) but not in total number of tender points (data not shown but available on request).

After adjusting for age, fat percentage, education level and marital status, the MANCOVA model informed the existence of significant differences between type of work groups; V = 0.605, F (12, 241) = 30.763, *P*<0.001. In particular comparisons, we found no differences in leisure-time physical activity between work groups at any intensity level (all, *P*>0.05). Comparisons for physical activity at home revealed that those women with a reproductive work had greater levels of light physical activity at home (mean difference=379.14, 95 % CI (175.64 to 582.64), *P*<0.001, Figure 1) and that there were no significant differences for the remaining comparisons of moderate (*P*=0.081) or vigorous (*P*=0.101) physical activity. Comparisons for physical activity at work (this is, house + work) revealed that those women with a productive work had greater levels of light physical activity at work (mean difference=448.52, 95 % CI (179.66 to 717.38), *P*=0.001, Figure 2) and that there were no significant differences for the remaining comparisons of moderate (*P*=0.142) or vigorous (*P*=0.920) physical activity. Comparisons for total physical activity (i.e., the addition of leisure time, house, and work) revealed that those women with a productive work had greater levels of light (mean difference=809.72, 95 % CI (535.91 to 1085.53), *P*<0.001, Figure 3) and moderate physical activity (mean difference=299.78, 95 % CI (97.31 to 502.25), *P*=0.004, Figure 4) but not of vigorous physical activity (*P*=0.089). No differences for sedentary behavior either on weekdays (*P*=0.308) or weekends were found (*P*=0.749).

For peer review only

DISCUSSION

In the present study, a number of differences were detected with regard to physical activity between women with fibromyalgia according to their type of work. In particular, women with only a reproductive work did more light physical activity at home. However, women with a productive work engage in more often in total physical activity (i.e., the addition of physical activity at leisure, home, and work) of light and moderate intensity. The remaining comparisons of physical activity in leisure time and sedentary behavior during weekdays and weekend, yielded not significant differences between both groups.

Our results revealed that both the group of women who did reproductive work and the group of women who also performed productive work obtained similar results in light, moderate and vigorous physical activity during their leisure time. According to a survey on sport habits in Spain [16], the lack of time is the major barrier to sport practice. Women, no matter if they work out of home or not, continue to be mostly responsible for the domestic work [3], what suggests that this work takes most of their leisure time. Moreover, according to Teniente [6], most women visualize themselves performing activities traditionally associated with their gender. Also, female sport is still living a situation of inequality, where sport is considered as a social activity related to men because they are the only ones to be supposed to have the physical characteristics to do it [6]. Some authors support that the main barriers to do physical activity for women are family care responsibilities [21,22] and leisure-time physical activities is reduced in women with family responsibilities [23]. In this regard, reproductive work depends on the family needs and requires dedication with no fixed schedule, turning leisure time into residual and variable time. Furthermore, those women who do productive work and also take care of the bulk of the unpaid work are in fact completing a double working day, so that their free time is reduced too [24]. Therefore, we hypothesized that no differences were observed between the two groups as both groups performed little physical activity due to lack of time because of family care or work responsibilities.

Light physical activity is the most common physical activity intensity in women with fibromyalgia [25]. In the present study, the group of women with fibromyalgia who did only

For peer review only

reproductive work showed higher levels of light physical activity (but not moderate or vigorous physical activity) at home. Spitze and Loscocco [26] pointed out that women who exclusively performed reproductive work spent more hours on housework because they had more time and they did not have to bear what is known as double day. This could be the reason why higher light physical activity during domestic work was observed among women who only did reproductive work, compared with the group who also did productive work. With regard to work, however, we found that women with a productive work engage more often in light physical activity at work; as the sum physical activity at both types of (reproductive and productive) work These findings could be explained by the so-called double day, this is, women with a reproductive work often perform most of the housework at their homes in addition to their work hours [27–29]. Indeed, reproductive work has been traditionally assigned to women and this is explained by gender differences [3,4,30], no matter whether women have incorporated to the productive sphere or not [30]. Furthermore, women who perform productive work are subject to their job demands, which prevent them from decreasing their performance or taking long breaks during their working hours [29]. Thus, also in agreement with the findings from Estévez-López et al [28], women who did productive work reported higher total physical activity levels compared with those who exclusively did reproductive work. Active commuting might be another potential cause of the physical activity increase in the group of women who did productive work [31], although others studies failed to find an association between work status and physical activity [23].

In the present study, women with fibromyalgia that only did reproductive work and those who additionally did a productive work engage in a similar time in sedentary behavior both in weekdays and on the weekend. Although previous studies have revealed that physically-demanding jobs are associated with increased pain and fatigue [32], the appropriate approach for this women would not be to make them more sedentary but, as stated by Galiano & Sañudo [33], to let them perform physical activity at such intensity that it leads to positive effects, but not as high as to increase the symptoms. In this regard, physical activity improves aspects such as quality of life of women with fibromyalgia [34], preventing problems derived from sedentary lifestyle. Therefore, we believe that an adaptation consisting in doing more sedentary work is not advisable for a woman with fibromyalgia. By contrast, it would be ideal to

For peer review only

make individual adaptations for every woman and her characteristics. However, work adaptations for people with fibromyalgia are not very frequent [35].

Although the cross-sectional design of the present study precludes to suggest the potential clinical implications of our findings, speculation might be done accordingly. As suggested by Kivimäki et al. [36], inability to work is often a consequence from fibromyalgia in women with this condition, so it would be recommendable to make adaptations of their working conditions. In relation to this, Henriksson, Liedberg & Gerdle [37] stated that adaptations made at work are key determinant of job maintenance. This is why these authors recommend establishing adaptation strategies such as modifying the tasks, reducing the working hours, determining their own schedule based on their state, working from home or adapting the working place. Therefore, in future research it would be advisable to go deeper into work adaptations and to assess the need for greater personalization. Interestingly, Palstam et al. [11] confirmed that people with fibromyalgia who performed productive work enjoyed better health than those not doing such work, especially in terms of pain, fatigue, stiffness, depression, physical aspects of quality of life and general state of health. For this reason, work was considered a relevant factor in the state of health of women with fibromyalgia [12], what suggests that they can continue working with no negative consequences for their condition as long as required adaptations are taken. It is important to bear in mind that to have a productive job is considered one of the main activities one's life, so adopting strategies to stay active would contribute to women with fibromyalgia personal development and self-esteem [27].

The conclusions of the present study must be taken bearing its limitations in mind. A cross-sectional design was used, so the results cannot be deemed as causal. The results cannot be extended to the rest of the Spanish population since this study has been conducted in Andalusia. Furthermore, self-administered questionnaires often provide misleading information in women with fibromyalgia [38]. However, objective tools such as accelerometers are not able to distinguish between whether the behavior is being done at leisure time, home or work. Thus, further research using objective data of

For peer review only

physical activity and sedentary behaviour at different settings is warranted when new engineering and data science developments allow to do so [39,40].

To conclude, our study revealed that, in general, differences were found in work and home-related (but not leisure-time) physical activity between women with fibromyalgia who exclusively performed reproductive work and those who also did productive work. In particular, women with only a reproductive work did more light physical activity at home. However, women with a productive work engage in more often in light physical activity at work (i.e., work + home) and total physical activity (i.e., the addition of physical activity at leisure, home and work) of light and moderate intensity. The remaining comparisons yielded not significant differences between both groups, which indicate similar physical activity in leisure time and sedentary behavior during weekdays and on the weekend. Given the cross-sectional design of this study, future longitudinal research is warranted in order to address the causality of the present findings. If they are corroborated, a robust suggestion would be that to have a productive work might promote higher physical activity levels in women with fibromyalgia. Also, it is well-known that having a job is key in people's personal development. Thus, a next step in research would be to find adaptations at work that help to balance to engage in bouts of physical activity of adequate length and intensity tailored to women with fibromyalgia capabilities.

Funding

This work was supported by the Spanish Ministries of Economy and Competitiviness (I+D+i DEP2010-15639; I+D+i DEP2013-40908-R) and the Spanish Ministry of Education (FPU15/00002). This study has been partially funded by the University of Granada, Plan Propio de Investigación 2016, Excellence actions: Units of Excellence; Unit of Excellence on Exercise and Health (UCEES), and by the Junta de Andalucía, Consejería de Conocimiento, Investigación y Universidades and European Regional Development Fund (ERDF), ref. SOMM17/6107/UGR.

For peer review only

Competing interests

The authors have declared no conflict of interest

Authors contributions

MJGR: Conception or design of the work, analysis and interpretation of data, and drafting the work for intellectual content; BGC: Analysis and interpretation of data and drafting the work for intellectual content; EAO: analysis and interpretation of data, and drafting the work for intellectual content; MBC: Analysis and interpretation of data and drafting the work for intellectual content; IGR: Acquisition of data and drafting the work for intellectual content; MDF: Conception or design of the work, acquisition of data, analysis and interpretation of data, and drafting the work for intellectual content; FEL: Conception or design of the work, acquisition of data, analysis and interpretation of data, and drafting the work for intellectual content.

REFERENCES

1 Carrasquer P, Torns T, Tejero E, *et al.* El Trabajo Reproductivo. *Pap Rev Sociol* 1998;**55**:95–114. doi:10.5565/rev/papers.1934

2 Benería L. Trabajo productivo/reproductivo, pobreza y políticas de conciliación. *Nómadas* 2006;:8–21.

3 Kan MY, Sullivan O, Gershuny J. Gender convergence in domestic work: Discerning the effects of interactional and institutional barriers from large-scale data. *Sociology* 2011;**45**:234–51. doi:10.1177/0038038510394014

4 Larrañaga, I., Arregui, B., & Arpal J. El trabajo reproductivo o doméstico. *Garceta Sanit* 2004;**18**:31–7.

5 Rohlfs I, Borrell C, Artazcoz L, *et al.* La importancia de la perspectiva de género en las encuestas de salud. *Gac Sanit* 2000;**14**:146–55. doi:10.1016/S0213-9111(00)71448-8

6 de Alba Teniente E. Experience of empowering women through sport. Case study of marathon

runners and ultra marathon runners. *Razón* 2017;**21**:281–94.

7 Pérez CB. Feminism, gender and reproductive work. *AusArt J Res Art* 2017;**5**:103–15. doi:10.1387/ausart.17782

8 Benlloch IM. Los efectos de las asimetrías de género en la salud de las mujeres. *Anu Psicol* 2003;**34**:253–66.

9 Mas AJ, Carmona L, Valverde M, *et al.* Prevalence and impact of fibromyalgia on function and quality of life in individuals from the general population: results from a nationwide study in Spain. *Clin Exp Rheumatol* 2008;**26**:519.

10 Wolfe F, Smythe HA, Yunus MB, *et al.* The American College of Rheumatology 1990 Criteria for the Classification of Fibromyalgia. Report of the Multicenter Criteria Committee. *Arthritis Rheum* 1990;**33**:160–72. doi:10.1002/art.1780330203

11 Palstam A, Bjersing JL, Mannerkorpi K. Which aspects of health differ between working and nonworking women with fibromyalgia? A cross-sectional study of work status and health. *BMC Public Health* 2012;**12**. doi:10.1186/1471-2458-12-1076

12 Reisine S, Fifield J, Walsh S, *et al.* Employment and health status changes among women with fibromyalgia: A five-year study. *Arthritis Care Res* 2008;**59**:1735–41. doi:10.1002/art.24309

13 Segura-Jimenez V, Borges-Cosic M, Soriano-Maldonado A, *et al.* Association of sedentary time and physical activity with pain, fatigue, and impact of fibromyalgia: the al-andalus study. *Scand J Med Sci Sports* 2017;**27**:83–92. doi:10.1111/sms.12630

14 Gavilán-Carrera B, Segura-Jiménez V, Mekary RA, *et al.* Substituting sedentary time with physical activity in fibromyalgia: association with quality of life and impact of the disease. The al-Ándalus project. *Arthritis Care Res (Hoboken)* 2018.

15 Ellingson LD, Shields MR, Stegner AJ, *et al.* Physical Activity, Sustained Sedentary Behavior, and Pain Modulation in Women With Fibromyalgia. *J Pain* 2012;**13**:195–206. doi:10.1016/j.jpain.2011.11.001

For peer review only

16 Ministerio de Educación C y D. Encuesta De Hábitos Deportivos 2015. 2015.

17 Segura-Jimenez V, Alvarez-Gallardo IC, Carbonell-Baeza A, *et al.* Fibromyalgia has a larger impact on physical health than on psychological health, yet both are markedly affected: The al-Andalus project. *Semin Arthritis Rheum* 2015;**44**:563–70. doi:10.1016/j.semarthrit.2014.09.010

18 Folstein MF, Folstein SE, McHugh PR. "Mini-mental state": a practical method for grading the cognitive state of patients for the clinician. *J Psychiatr Res* 1975;**12**:189–98.

19 Mannerkorpi K, Hernelid C. Leisure time physical activity instrument and physical activity at home and work instrument. Development, face validity, construct validity and test-retest reliability for subjects with fibromyalgia. *Disabil Rehabil* 2005;**27**:695–701. doi:10.1080/09638280400009063

20 Rosenberg DE, Norman GJ, Wagner N, *et al.* Reliability and validity of the Sedentary Behavior Questionnaire (SBQ) for adults. *J Phys Act Health* 2010;**7**:697–705. doi:10.1123/jpah.7.6.697

21 Martínez del Castillo J, Martin Rodriguez M, Hierro Pinés D del, *et al.* Barriers to the physical activity in the adult and alternative women of conciliation. *Act física y Deport Cienc y profesión* 2008;:12–24.

22 Vázquez Gómez B. Mujeres y actividades físico-deportivas (Investigaciones en Ciencias del Deporte, nº35). *Madrid Cons Super Deport* 2002.

23 Bopp M, Child S, Campbell M. Factors Associated with Active Commuting to Work Among Women. *Women Health* 2014;**54**:212–31. doi:10.1080/03630242.2014.883663

24 Vázquez Gómez B. Nuevos Retos para el Deporte y las Mujeres en el Siglo XXI. In: *Actas del Congreso Mujer y Deporte, Bilbao*. 2001.

25 Segura-Jiménez V, Álvarez-Gallardo IC, Estévez-López F, *et al.* Differences in Sedentary Time and Physical Activity Between Female Patients With Fibromyalgia and Healthy Controls: The al-Ándalus Project. *Arthritis Rheumatol* 2015;**67**:3047–57. doi:10.1002/art.39252

26 Spitze G, Loscocco KA. The labor of sisyphus?: women's and men's reactions to housework.

For peer review only

Soc Sci Q 2000;**81**:1087–101.

27 Briones-Vozmediano E, Ronda-Pérez E, Vives-Cases C. Percepciones de pacientes con fibromialgia sobre el impacto de la enfermedad en el ámbito laboral. *Aten Primaria* 2015;**47**:205–12. doi:10.1016/j.aprim.2014.06.002

28 Estevez-Lopez F. *Fibromyalgia: adapted, positive, and fit people: Breaking stereotypes!* 2018.

29 Gómez Ortíz V. Estrés y Salud en mujeres que desempeñan múltiples roles. *Av en Psicol Latinoam* 2004;**22**:117–28.

30 Mazzei C. Producción y reproducción: la mujer y la división socio-sexual del trabajo. *Rumbos TS, Univ Cent Chile* 2013;**VII**, **N°8**:128–42.

31 Csizmadi I, Lo Siou G, Friedenreich CM, *et al.* Hours spent and energy expended in physical activity domains: Results from The Tomorrow Project cohort in Alberta, Canada. *Int J Behav Nutr Phys Act* 2011;**8**. doi:10.1186/1479-5868-8-110

32 Dailey DL, Keffala VJ, Sluka KA. Do cognitive and physical fatigue tasks enhance pain, cognitive fatigue, and physical fatigue in people with fibromyalgia? *Arthritis Care Res* 2015;**67**:288–96. doi:10.1002/acr.22417

33 Galiano OD, Sañudo CB. Aplicación del ejercicio físico como terapia en medicina del trabajo para pacientes con fibromialgia. *Med Segur Trab (Madr)* 2013;**59**:310–21. doi:10.4321/S0465-546X2013000300003

34 Cadenas-Sanchez C, Ruiz-Ruiz J. Effect of a physical activity programme in patients with fibromyalgia: A systematic review. *Med Clin (Barc)* 2014;**143**:548–53. doi:10.1016/j.medcli.2013.12.010

35 Bossema ER, Kool MB, Cornet D, *et al.* Characteristics of suitable work from the perspective of patients with fibromyalgia. *Rheumatology* 2012;**51**:311–8. doi:10.1093/rheumatology/ker312

36 Kivimäki M, Leino-Arjas P, Kaila-Kangas L, *et al.* Increased absence due to sickness among employees with fibromyalgia. *Ann Rheum Dis* 2007;**66**:65–9.

For peer review only

37 Henriksson CM, Liedberg GM, Gerdle B. Women with fibromyalgia: work and rehabilitation. *Disabil Rehabil* 2005;**27**:685–94.

38 Segura-Jimenez V, Alvarez-Gallardo IC, Romero-zurita A, *et al.* Comparison of Physical Activity Using Questionnaires (Leisure Time Physical Activity Instrument and Physical Activity at Home and Work Instrument) and Accelerometry in Fibromyalgia Patients : The al-Ándalus Project. *Arch Phys Med Rehabil* Published Online First: 2014. doi:10.1016/j.apmr.2014.05.015

39 Estévez-López F, Martinez-Tellez B, Ruiz JR. Physical fitness and cancer. *Lancet Oncol* 2017;**18**.

40 Kerr J, Anderson C, Lippman SM. Physical activity, sedentary behaviour, diet, and cancer: an update and emerging new evidence. *Lancet Oncol* 2017;**18**:e457--e471.

For peer review only

Table 1. Sociodemographic characteristics of the participants (*n*=258)

Educational level		
Incomplete studies	25	(9.7)
Primary school	132	(51.2)
Secondary school	65	(25.2)
University degree	36	(14.0)
Marital status		
Married	201	(77.9)
Single	14	(5.4)
Separated/divorced	26	(10.1)
Widowed	17	(6.6)
Current working status		
Reproductive work	143	(55.4)
Productive work	115	(44.6)
	Mean	(SD)
Age	51.4	(7.9)
Fat percentage (%)	40.2	(7.3)
Total number of tender points	16.6	(2.0)
Leisure-time PA (LTPAI, min/week)		
Light (< 3 METs)	178.9	(143.0)
Moderate (3-6 METs)	62.8	(100.3)
Vigorous (> 6 METs)	16.5	(43.6)
PA at home (PAHWI, min/week)		
Light (< 3 METs)	1188.8	(786.2)
Moderate (3-6 METs)	544.4	(521.0)
Vigorous (> 6 METs)	70.4	(249.2)
PA at work (reproductive and productive, min/week)		
Light	1542.5	(1003.2)
Moderate	607.2	(553.1)
Vigorous	86.9	(258.8)
Total sedentary time (SBQ)		
Monday to Friday (min/weekday)	533.1	(261.2)
During the weekend (min/weekend day)	566.2	(257.6)

SD, standard deviation; PA, physical activity; METs, metabolic index measurement unit; LTPAI, Leisure-Time Physical Activity Instrument; PAHWI, Physical Activity at Home and Work Instrument; SBQ, Sedentary Behavior Questionnaire.

For peer review only

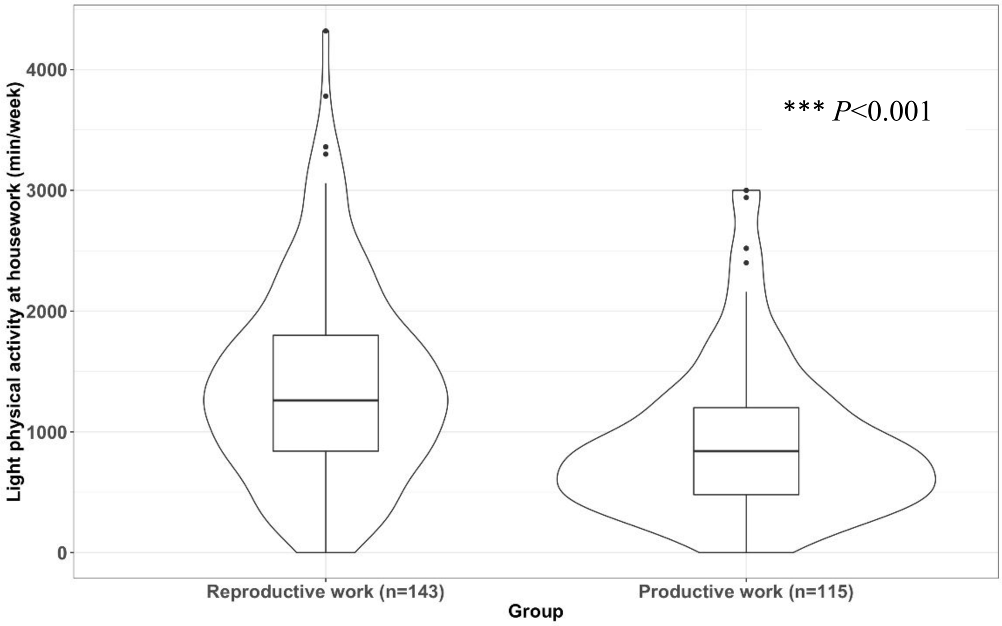

Figure 1. Differences in physical activity at home of light intensity between women with fibromyalgia who only performed reproductive work and those who also performed productive work.

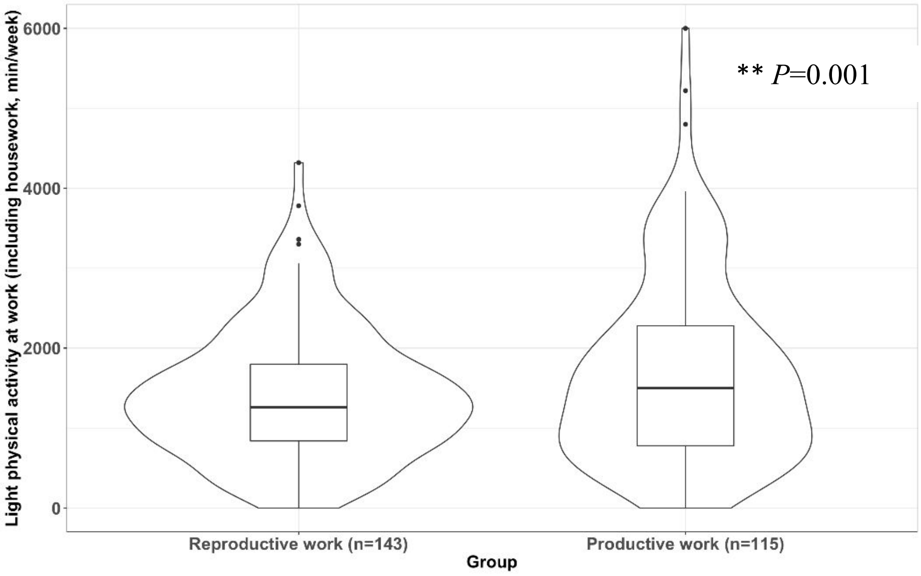

Figure 2. Differences in physical activity work (i.e., house + work) of light intensity between women with fibromyalgia who only performed reproductive work and those who also performed productive work.

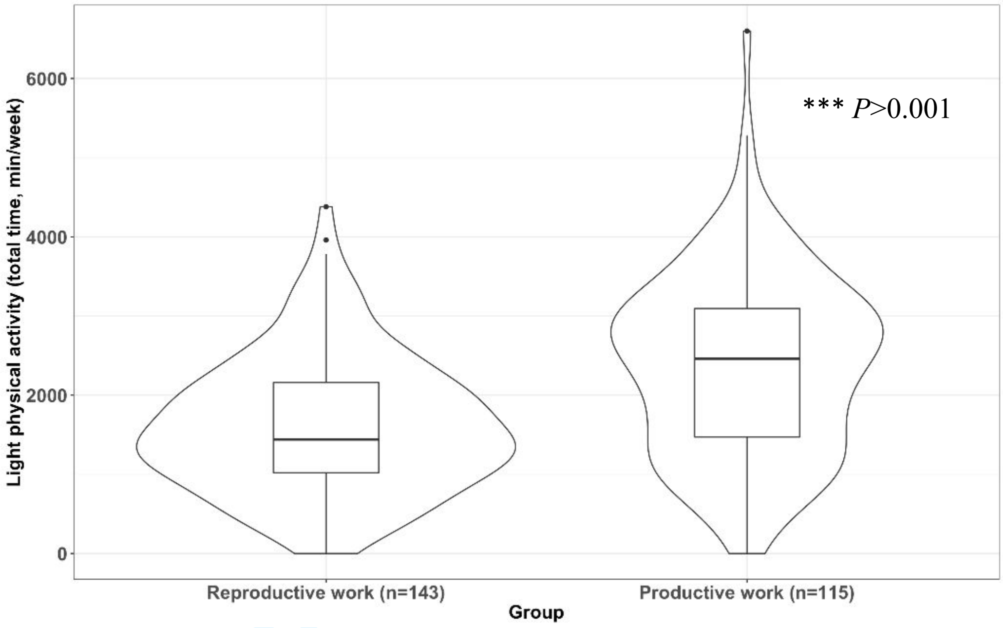

Figure 3. Differences in total physical activity (i.e., the addition of leisure time, house, and work) of light intensity between women with fibromyalgia who only performed reproductive work and those who also performed productive work.

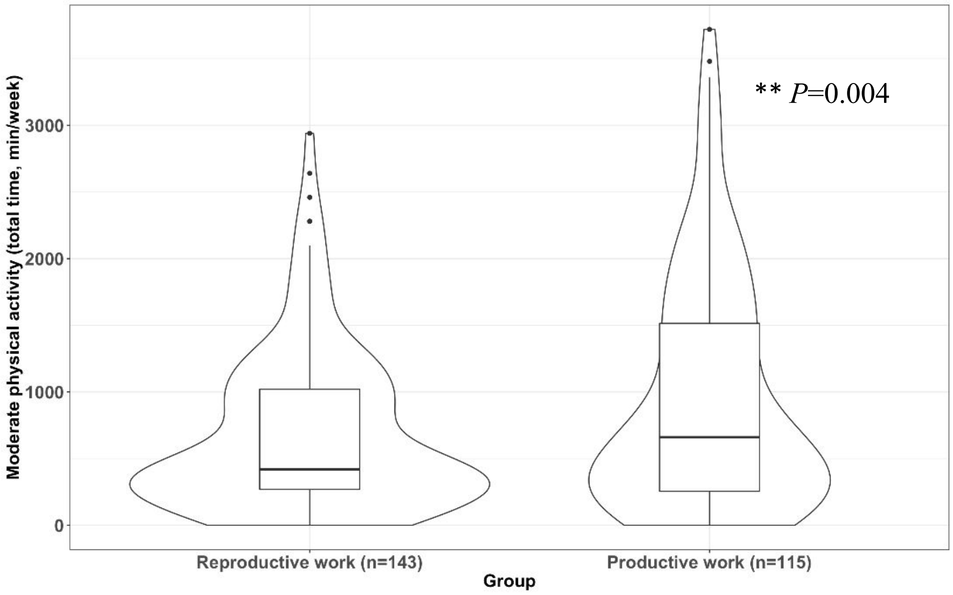

Figure 4. Differences in total physical activity (i.e., the addition of leisure time, house, and work) of moderate intensity between women with fibromyalgia who only performed reproductive work and those who also performed productive work

BMJ Open

Is type of work associated with physical activity and sedentary behavior in women with fibromyalgia? A cross-sectional study from the al-Ándalus project.

Journal:	*BMJ Open*
Manuscript ID	bmjopen-2019-034697.R1
Article Type:	Original research
Date Submitted by the Author:	20-Jan-2020
Complete List of Authors:	Girela-Rejón, Maria José; University of Granada, Department of Didactic of Corporal Expression, Faculty of Education Sciences<br>Gavilán Carrera, Blanca; University of Granada, Department of Physical Education and Sport, Faculty of Sport Sciences; Sport and Health University Research Institute (iMUDS)<br>Aparicio-Ortega, Esther; University of Granada, Department of Physical Education and Sport, Faculty of Sport Sciences<br>Borges-Cosic, Milkana; University of Granada, Department of Physical Education and Sport, Faculty of Sport Sciences; Sport and Health University Research Institute (iMUDS)<br>García-Rodríguez, Inmaculada C; University of Granada, Department of Physical Education and Sport, Faculty of Sport Sciences<br>Delgado-Fernández, Manuel; University of Granada, Department of Physical Education and Sport, Faculty of Sport Sciences; Sport and Health University Research Institute (iMUDS)<br>Estévez-López, Fernando; Erasmus MC University Medical Center, Department of Child and Adolescent Psychiatry/Psychology
**Primary Subject Heading**:	Rheumatology
Secondary Subject Heading:	Sports and exercise medicine, Rehabilitation medicine
Keywords:	RHEUMATOLOGY, Pain management < ANAESTHETICS, SPORTS MEDICINE

SCHOLARONE™
Manuscripts

For peer review only - http://bmjopen.bmj.com/site/about/guidelines.xhtml

I, the Submitting Author has the right to grant and does grant on behalf of all authors of the Work (as defined in the below author licence), an exclusive licence and/or a non-exclusive licence for contributions from authors who are: i) UK Crown employees; ii) where BMJ has agreed a CC-BY licence shall apply, and/or iii) in accordance with the terms applicable for US Federal Government officers or employees acting as part of their official duties; on a worldwide, perpetual, irrevocable, royalty-free basis to BMJ Publishing Group Ltd ("BMJ") its licensees and where the relevant Journal is co-owned by BMJ to the co-owners of the Journal, to publish the Work in this journal and any other BMJ products and to exploit all rights, as set out in our licence.

The Submitting Author accepts and understands that any supply made under these terms is made by BMJ to the Submitting Author unless you are acting as an employee on behalf of your employer or a postgraduate student of an affiliated institution which is paying any applicable article publishing charge ("APC") for Open Access articles. Where the Submitting Author wishes to make the Work available on an Open Access basis (and intends to pay the relevant APC), the terms of reuse of such Open Access shall be governed by a Creative Commons licence – details of these licences and which Creative Commons licence will apply to this Work are set out in our licence referred to above.

Other than as permitted in any relevant BMJ Author's Self Archiving Policies, I confirm this Work has not been accepted for publication elsewhere, is not being considered for publication elsewhere and does not duplicate material already published. I confirm all authors consent to publication of this Work and authorise the granting of this licence.

For peer review only

Title: Is type of work associated with physical activity and sedentary behavior in women with fibromyalgia? A cross-sectional study from the al-Ándalus project.

Authors:

Maria José Girela-Rejón, PhD[1*], Blanca Gavilán-Carrera, MSc[2,3*], Esther Aparicio-Ortega, MSc[2], Milkana Borges-Cosic, PhD[2,3], Inmaculada C García-Rodríguez, MSc[2,3], Manuel Delgado-Fernández, PhD[2,3], Fernando Estévez-López, PhD[4].

Affiliations:

1 Department of Didactic of Corporal Expression, Faculty of Education Sciences, University of Granada, Spain.

2 Department of Physical Education and Sport, Faculty of Sport Sciences, University of Granada, Granada, Spain.

3 Sport and Health University Research Institute (iMUDS)

4 Department of Child and Adolescent Psychiatry/Psychology, Erasmus MC University Medical Center, Rotterdam, the Netherlands.

*These authors equally contributed to this work

Corresponding author

Fernando Estévez-López

Department of Child and Adolescent Psychiatry/Psychology, Erasmus MC University Medical Center, Rotterdam, the Netherlands

Email: f.estevez-lopez@erasmusmc.nl

Keywords: Chronic pain; Lifestyle; Rheumatic and musculoskeletal diseases; Women's health

For peer review only

Word count: 3625

ABSTRACT

Objectives: To analyze the association between the type of work (productive vs. reproductive work) and the levels of physical activity and sedentary behavior in women with fibromyalgia.

Method: This cross-sectional study involved 258 women with fibromyalgia from southern Spain. Of them, 55% performed reproductive work (unpaid, associated with care giving and domestic roles) exclusively, while 45% had a productive job (remunerated, that results in good or services). Physical activity of light, moderate and vigorous intensity in the leisure time, at home, at work, and total were measured through the Leisure Time Physical Activity Instrument (LTPAI) and with the Physical Activity at Home and Work Instrument (PAHWI), respectively. Sedentary behavior was measured by the Sedentary Behavior Questionnaire (SBQ).

Results: After adjusting for age, fat percentage, education level and marital status, the MANCOVA model informed the existence of significant differences between type of work groups ($P<0.001$). Women with a productive work engaged in more light physical activity at work (mean difference=448.52 min; 95 % CI 179.66 to 717.38; $P=0.001$), and total physical activity of light (809.72 min; 535.91 to 1085.53; $P<0.001$) and moderate (299.78; 97.31 to 502.25; $P=0.004$) intensity. Women with a reproductive work engaged in more light physical activity at home (379.14; 175.64 to 582.64; $P<0.001$). Leisure time physical activity and sedentary behavior was similar in both groups ($P>0.05$ for all comparisons).

Conclusions: Women with a productive work had greater levels of physical activity compared to those who only did reproductive work, except for physical activity at home. To have a productive work might facilitate that women with fibromyalgia move towards a more active lifestyle.

For peer review only

Strengths and limitations of this study

- The questionnaires used to assess physical activity are able to distinguish whether the behavior is being done at leisure time, home or work.
- A large sample of patients with fibromyalgia representative of southern Spain was included.
- The cross-sectional impedes determination of causal relationships.
- Self-administered questionnaires could provide misleading information in women with fibromyalgia.

For peer review only

INTRODUCTION

Reproductive work refers to all those tasks whose purpose is to take care and ensure the wellness of the family and household, reason why it is also called domestic work. Its main characteristics are: not to be remunerated by salary, to be mainly female work and to stay invisible [1]. Nevertheless, the economically and socially recognized work is the productive work (goods and services), which refers to all those human activities that have an exchange value and lead to financial remuneration [1,2].

The society in general transmits values and rules that shape female and male identities, so that work is differentiated by gender, men being more oriented to productive work and women being typically the main responsible for domestic work and family care, called reproductive work [3–5]. The majority of women imagine themselves performing activities associated with their gender and live with the aim of meeting the expectations related with their maternal role. Therefore, the major difference between genders has been and remains to be the reproductive work [6,7]. The lower visibility and social recognition of reproductive work also entails less economic independence as it is unpaid [1]. Despite women's increasing participation in the work market, this means to assume that women do the so-called double working day, in which they face the difficulty of combining productive and reproductive work [3,8]

Fibromyalgia is a condition that affects 2.4% of the Spanish population, being more frequent among women than among men [9]. Fibromyalgia is heterogeneous [10] characterized by widespread musculoskeletal pain of unknown origin, as well as fatigue, joint stiffness and sleep problems, among others [11,12], what has negative consequences on their ability to work [13]. The idea that health problems are not randomly distributed, but influenced by gender inequality is becoming increasingly popular, and the social context of women with fibromyalgia is a society that assigns them the caring role (either for children or elderly). Thus, the family caring responsibilities and the double working day that some of these women perform (remunerated work and housework) are factors that also affect their health status [8]. For instance, those women who perform productive work experience better health than those who do not do that kind of work in terms of pain, fatigue, and depression [14]. Among the factors that could positively influence fibromyalgia symptoms, there is evidence that a higher level physical activity of different intensities and lower levels of sedentary behavior are associated with better symptomatology [15–17]. Unfortunately, the difficulties when reconciling productive and reproductive works could lead to a lack of time for those women [7], which is a potential barrier not to practice physical activity [18]. This potential relationship between work type and physical activity, which may determine health in this group of patients, is, however, not well-characterized yet.

Therefore, the aim of the present study was to analyze the association between the type of work (productive vs. reproductive), physical activity intensity levels (in leisure time, home, work, and total), and sedentary behavior in women with fibromyalgia.

METHOD

Design and participants

This study belongs to al-Ándalus project, in which a representative sample of women with fibromyalgia from Andalusia (Spain) was examined [19]. Data from the participants were collected between 1 November 2011 and 31 December 2013. Associations from the eight Andalusian provinces were contacted and other participants were recruited through e-mail, letter, telephone or the university

For peer review only

website. A total of 646 individuals with fibromyalgia were interested in participating in the study. The inclusion criteria for the present study were: i) to have been diagnosed with fibromyalgia; ii) to meet the 1990 ACR criteria [11]; iii) not to suffer from any acute or terminal illness, or serious cognitive impairment; iv) to be a woman. The al-Ándalus project protocol was approved by the ethics committee of Virgen de las Nieves Hospital (Granada, Spain) with register number: 15/11/2013-N72. The guidelines of the Declaration of Helsinki (modified in 2000) were followed. This study also follows the standards for reporting observational studies stated in the STROBE initiative (Strengthening the Reporting of Observational studies in Epidemiology checklist; Supplementary Table S1).

Procedure and instruments

The measurements were conducted in two non-consecutive days. On the first day, the participants signed an informed consent, completed a questionnaire on sociodemographic data, underwent the Mini-Mental State Examination (MMSE) [20] and were examined for tender points and body composition. Between days 1 and 2, they completed the following questionnaires at home: Leisure Time Physical Activity Instrument (LTPAI), Physical Activity at Home and Work Instrument (PAHWI) [21] and Sedentary Behavior Questionnaire (SBQ) [22]. On the second day, participants returned to the laboratory and questionnaires were checked by the research group.

Sociodemographic data

The sociodemographic data were obtained through a questionnaire that included items related to educational level, marital status and working status. Working status was classified as (i) patients with that only have a reproductive work (this is, unpaid and associated with care giving and domestic roles) or (ii) patients that also have a productive work (this is remunerated, that results in good or services). Women classified in productive work could do reproductive work at the same time.

Mini-Mental State Examination

For peer review only

It was used to detect severe cognitive impairment. Its score ranges between 0 and 30, and a score under 10 was considered as cognitive impairment. It is a short test for cognitive assessment that includes questions to evaluate five areas of the cognitive function: orientation, short-term memory, attention, concentration, long-term memory and language [20].

Tender points

The 18 tender points were assessed according to ACR guidelines [11] for the diagnosis of fibromyalgia using a standardized pressure algometer (FPK 20; Wagner Instruments, Greenwich, CT, USA). Increasing pressure was applied with the algometer on the selected area and the patients were requested to inform when they started to feel pain. The tender points were classified as positive when pain appeared at a pressure of 4kg/cm^2 or lower. The same procedure was repeated twice and the mean was calculated. The total number of positive tender points was recorded for every patient. The participants needed to have a minimum of 11 positive points in order to confirm the diagnosis of fibromyalgia.

Fat percentage

The fat percentage was measured by means of a portable bioelectrical impedance analyzer with eight tactile electrodes (InBody R20, Biospace, Seoul, Korea). The participants were requested to refrain from taking a shower, performing intense physical activity or eating in the two hours prior to the measurement. They were asked to wear only underwear and no metal objects that could interfere in the measurement.

Leisure Time Physical Activity Instrument

The LTPAI is an instrument that has shown satisfactory reliability among patients with fibromyalgia [21]. It comprises four items with three intensity levels: light, moderate and vigorous. The participants were requested to indicate the number of hours a week they had performed leisure physical activity during the previous 4 weeks. The scale can be simplified in the following levels: i) 0.5 to 1.5

For peer review only

hours per week, ii) 2 to 4 hours per week, iii) > 4 hours per week. When a participant selected levels 1 or 2, the middle range value was used for the total score. When level 3 was selected, she was asked to provide the number of hours. When no level was selected, the number of hours was considered to be 0. The number of hours indicated by the participants for every intensity category was added up to obtain the leisure-time physical activity level during one week.

Physical Activity at Home and Work Instrument

The PAHWI questionnaire [21], comprises seven items with three categories for work performed at home (light, moderate and vigorous activity) and four categories for work performed out of home (sedentary, light, moderate and vigorous activity). The participants were asked to provide the average number of hours per week that they had spent on activities of each category in the previous four weeks. The hours of each category were added up to obtain the total score.

Total physical activity

Although the LTPAI and the PAHWI were administered separately, they both belong to a more complex questionnaire that covers all physical activity performed during the day: leisure time, housework, and work. The three categories (light, moderate and vigorous) of both questionnaires were summed up to obtain the total number of minutes of physical activity per week. The final result was divided by 7 to get the total number of minutes of physical activity per day. This custom in-house approach has been used previously (e.g., [23]).

Sedentary Behavior Questionnaire

The SBQ [22] informs about the time spent on 11 sedentary behaviors (watching television, sitting while eating, lying and resting, sitting while playing computer/video games, sitting while listening to music, sitting and talking on the phone, doing paperwork or office work, sitting and reading, playing a musical instrument, doing arts and crafts, sitting and driving/travelling in a car, bus, or train). The 11 items were completed separately for weekdays and weekend days. Response options were

For peer review only

"none", "15 minutes or less", "30 minutes", "1 hour", "2 hours", "3 hours", "4 hours", "5 hours", or "6 hours or more". The time spent on each behavior was converted into hours (this is 15 minutes=0.25 hours). Hours per day for each item were summed separately for weekday and weekend days for the total scores of sedentary behaviors. For the summary variables of total hours/day spent in sedentary behaviors (weekday and weekend) and total sedentary hours/week, responses higher than 24 hours/day were truncated to 24 hours/ day.

Statistical analysis

Normal distribution was assumed due to large sample size. Before main analyses and in order to identify potential confounders, the age, fat percentage, total number of tender points, education level (i.e., unfinished/primary studies or secondary/vocational/university studies) and marital status (i.e., currently married or unmarried) of participants with only reproductive work and those with a productive work were compared using unpaired samples t-test or χ^2 tests. Given that significant differences between groups emerged for all these variables (with the exception of total number of tender points), age, fat percentage, education level, and marital status were included as covariates in all the analyses described below. Unadjusted analyses and analyses additionally accounting for disease severity assessed by the Fibromyalgia Impact Questionnaire [24] are included as supplementary material (supplementary tables 2 and 3).

A one-way multivariate analysis of covariance (MANCOVA) was conducted to compare the mean scores of women with fibromyalgia according to their type of work (i.e., participants with only reproductive work and those with a productive work) on (i) leisure time physical activity (of light, moderate, and vigorous physical activity), (ii) physical activity at home (of light, moderate, and vigorous physical activity), (iii) physical activity at work (i.e., the addition of housework and work) of light, moderate, and vigorous physical activity, (iv) total physical activity (i.e., the addition of leisure time, housework, and work) of light, moderate, and vigorous physical activity, (v) sedentary behavior during weekdays and (vi) sedentary behavior on the weekend. MANCOVA allows dependent variables to be correlated and is more powerful than ANCOVA for detecting group differences. Statistical

For peer review only

significance was set at *P*<0.05 in all analyses. The Statistical Package for Social Sciences software (IBM SPSS, version 22.0) was used. Additionally, to illustrate our significant results, violin plots were created using R package ggplot2.

PATIENT AND PUBLIC INVOLVEMENT

Patients were not involved in the development of the research question of this study. During the recruitment process, patient associations from the eight Andalusian provinces were approached to obtain collaboration agreements and to facilitate field access. Patient associations supported recruitment by disseminating advertisement for study participation. Individual patients were also reached and informed about the study aims by the research team through e-mail, letter, telephone or the university website. During the conduct of the study, patients' previous experiences in the initial phases of the al-Ándalus project were considered to create the assessment protocol. The assessment of different outcomes was planned so patients have enough time to fill questionnaires, rest, and recover after exhausting tests. Several talks have been carried out in the associations, at conferences and in media to disseminate the results of the al-Ándalus project.

RESULTS

The flowchart of the participants included in the present study is shown in Figure 1. The final sample was composed of 258 women with fibromyalgia. Table 1 shows the sample characteristics. The majority of the participants were married (77.9%) and had low educational level (incomplete studies 9.7%; primary school 51.2%; secondary school 25.2%). 55.4% of the women performed reproductive work, while 44.6% performed productive work. The participants' mean age was 51 years old and their mean fat percentage was 40.2%. Significant differences emerged between groups (productive vs. reproductive) on age, body fat percentage, education level and marital status (all, P's $\leq$ 0.001) but not in total number of tender points (data not shown but available on request).

For peer review only

After adjusting for age, fat percentage, education level and marital status, the MANCOVA model informed the existence of significant differences between type of work groups; V = 0.605, $F(12, 241) = 30.763$, $P<0.001$. In particular comparisons, we found no differences in leisure-time physical activity between work groups at any intensity level (all, $P>0.05$). Comparisons for physical activity at home revealed that those women with a reproductive work had greater levels of light physical activity at home (mean difference=379.14, 95 % CI (175.64 to 582.64), $P<0.001$, Figure 2) and that there were no significant differences for the remaining comparisons of moderate ($P=0.081$) or vigorous ($P=0.101$) physical activity. Comparisons for physical activity at work (this is, house + work) revealed that those women with a productive work had greater levels of light physical activity at work (mean difference=448.52, 95 % CI (179.66 to 717.38), $P=0.001$, Figure 3) and that there were no significant differences for the remaining comparisons of moderate ($P=0.142$) or vigorous ($P=0.920$) physical activity. Comparisons for total physical activity (i.e., the addition of leisure time, house, and work) revealed that those women with a productive work had greater levels of light (mean difference=809.72, 95 % CI (535.91 to 1085.53), $P<0.001$, Figure 4) and moderate physical activity (mean difference=299.78, 95 % CI (97.31 to 502.25), $P=0.004$, Figure 5) but not of vigorous physical activity ($P=0.089$). No differences for sedentary behavior either on weekdays ($P=0.308$) or weekends were found ($P=0.749$). Sensitivity analyses additionally considering disease severity did not alter the results (supplementary tables 2 and 3).

DISCUSSION

In the present study, a number of differences were detected with regard to physical activity between women with fibromyalgia according to their type of work. In particular, women with only a reproductive work did more light physical activity at home. However, women with a productive work engage in more often in total physical activity (i.e., the addition of physical activity at leisure, home, and work) of light and moderate intensity. The remaining comparisons of physical activity in leisure time and sedentary behavior during weekdays and weekend, yielded not significant differences between both groups.

For peer review only

Our results revealed that both the group of women who did reproductive work and the group of women who also performed productive work obtained similar results in light, moderate and vigorous physical activity during their leisure time. According to a survey on sport habits in Spain [18], the lack of time is the major barrier to sport practice. Women, no matter if they work out of home or not, continue to be mostly responsible for the domestic work [3], what suggests that this work takes most of their leisure time. Moreover, according to Teniente [6], most women visualize themselves performing activities traditionally associated with their gender. Also, female sport is still living a situation of inequality, where sport is considered as a social activity related to men because they are the only ones to be supposed to have the physical characteristics to do it [6]. Some authors support that the main barriers to do physical activity for women are family care responsibilities [25,26] and leisure-time physical activities is reduced in women with family responsibilities [27]. In this regard, reproductive work depends on the family needs and requires dedication with no fixed schedule, turning leisure time into residual and variable time. Furthermore, those women who do productive work and also take care of the bulk of the unpaid work are in fact completing a double working day, so that their free time is reduced too [28]. Therefore, we hypothesized that no differences were observed between the two groups as both groups performed little physical activity due to lack of time because of family care or work responsibilities.

Light physical activity is the most common physical activity intensity in women with fibromyalgia [29]. In the present study, the group of women with fibromyalgia who did only reproductive work showed higher levels of light physical activity (but not moderate or vigorous physical activity) at home. Spitze and Loscocco [30] pointed out that women who exclusively performed reproductive work spent more hours on housework because they had more time and they did not have to bear what is known as double day. This could be the reason why higher light physical activity during domestic work was observed among women who only did reproductive work, compared with the group who also did productive work. With regard to work, however, we found that women with a productive work engage more often in light physical activity at work; as the sum physical activity at both types of (reproductive and productive) work. These findings could be explained by the so-called double day,

For peer review only

this is, women with a reproductive work often perform most of the housework at their homes in addition to their work hours [31–33]. Indeed, reproductive work has been traditionally assigned to women and this is explained by gender differences [3,4,34], no matter whether women have incorporated to the productive sphere or not [34]. Furthermore, women who perform productive work are subject to their job demands, which prevent them from decreasing their performance or taking long breaks during their working hours [33]. Thus, also in agreement with the findings from Álvarez-Gallardo et al. [35], women who did productive work reported higher total physical activity levels compared with those who exclusively did reproductive work. Active commuting might be another potential cause of the physical activity increase in the group of women who did productive work [36], although others studies failed to find an association between work status and physical activity [27].

In the present study, women with fibromyalgia that only did reproductive work and those who additionally did a productive work engage in a similar time in sedentary behavior both in weekdays and on the weekend. Although previous studies have revealed that physically-demanding jobs are associated with increased pain and fatigue [37], the appropriate approach for this women would not be to make them more sedentary but, as stated by Galiano & Sañudo [38], to let them perform physical activity at such intensity that it leads to positive effects, but not as high as to increase the symptoms. In this regard, physical activity improves aspects such as quality of life of women with fibromyalgia [39], preventing problems derived from sedentary lifestyle. Therefore, we believe that an adaptation consisting in doing more sedentary work is not advisable for a woman with fibromyalgia. By contrast, it would be ideal to make individual adaptations for every woman and her characteristics. However, work adaptations for people with fibromyalgia are not very frequent [40].

Although the cross-sectional design of the present study precludes to suggest the potential clinical implications of our findings, speculation might be done accordingly. As suggested by Kivimäki et al. [41], inability to work is often a consequence from fibromyalgia in women with this condition, so it would be recommendable to make adaptations of their working conditions. In relation to this, Henriksson, Liedberg & Gerdle [42] stated that adaptations made at work are key determinant of job

For peer review only

maintenance. This is why these authors recommend establishing adaptation strategies such as modifying the tasks, reducing the working hours, determining their own schedule based on their state, working from home or adapting the working place. Therefore, in future research it would be advisable to go deeper into work adaptations and to assess the need for greater personalization. Interestingly, Palstam et al. [13] confirmed that people with fibromyalgia who performed productive work enjoyed better health than those not doing such work, especially in terms of pain, fatigue, stiffness, depression, physical aspects of quality of life and general state of health. For this reason, work was considered a relevant factor in the state of health of women with fibromyalgia [14], what suggests that they can continue working with no negative consequences for their condition as long as required adaptations are taken. It is important to bear in mind that to have a productive job is considered one of the main activities one's life, so adopting strategies to stay active would contribute to women with fibromyalgia personal development and self-esteem [31].

The conclusions of the present study must be taken bearing its limitations in mind. A cross-sectional design was used, so the results cannot be deemed as causal. The results cannot be extended to the rest of the Spanish population since this study has been conducted in Andalusia. Furthermore, self-administered questionnaires are often seen as providers of misleading information in women with fibromyalgia [43]. However, objective tools such as accelerometers are not able to distinguish between whether the behavior is being done at leisure time, home or work. Thus, further research using objective data of physical activity and sedentary behaviour at different settings is warranted when new engineering and data science developments allow to do so [44,45]. While these developments are not performed, subjective and objective information may be better seen as complementary [23,46]. Other limitation is that type of activity (e.g., mostly either sitting, standing, walking, or physical labour) performed during work was not considered in the present study. For instance, previous studies have observed that different type of jobs (i.e., mostly either sitting, standing, walking, or physical labour) are concurrently associated with levels of physical activity and sedentary behaviour [47,48]. Thus, future research should account for type of activity during (productive and reproductive) work to comprehensively understand behavioural patterns of people with fibromyalgia at work.

For peer review only

To conclude, our study revealed that, in general, differences were found in work and home-related (but not leisure-time) physical activity between women with fibromyalgia who exclusively performed reproductive work and those who also did productive work. In particular, women with only a reproductive work did more light physical activity at home. However, women with a productive work engage in more often in light physical activity at work (i.e., work + home) and total physical activity (i.e., the addition of physical activity at leisure, home and work) of light and moderate intensity. The remaining comparisons yielded not significant differences between both groups, which indicate similar physical activity in leisure time and sedentary behavior during weekdays and on the weekend. Given the cross-sectional design of this study, future longitudinal research is warranted in order to address the causality of the present findings. If they are corroborated, a robust suggestion would be that to have a productive work might promote higher physical activity levels in women with fibromyalgia. Also, it is well-known that having a job is key in people's personal development. Thus, a next step in research would be to find adaptations at work that help to balance to engage in bouts of physical activity of adequate length and intensity tailored to women with fibromyalgia capabilities.

Funding

This work was supported by the Spanish Ministries of Economy and Competitiveness (I+D+i DEP2010-15639; I+D+i DEP2013-40908-R) and the Spanish Ministry of Education (FPU15/00002). This study has been partially funded by the University of Granada, Plan Propio de Investigación 2016, Excellence actions: Units of Excellence; Unit of Excellence on Exercise and Health (UCEES), and by the Junta de Andalucía, Consejería de Conocimiento, Investigación y Universidades and European Regional Development Fund (ERDF), ref. SOMM17/6107/UGR. FE-L has received funding from the European Union's Horizon 2020 research and innovation programme under the Marie Skłodowska-Curie grant agreement no. 707404. The funders of this study did not have any role in the study design, data collection and analyses, decision to publish or preparation of the manuscript.

For peer review only

Competing interests

The authors have declared no conflict of interest

Authors contributions

MJGR: Conception or design of the work, analysis and interpretation of data, and drafting the work for intellectual content; BGC: Analysis and interpretation of data and drafting the work for intellectual content; EAO: analysis and interpretation of data, and drafting the work for intellectual content; MBC: Analysis and interpretation of data and drafting the work for intellectual content; IGR: Acquisition of data and drafting the work for intellectual content; MDF: Conception or design of the work, acquisition of data, analysis and interpretation of data, and drafting the work for intellectual content; FEL: Conception or design of the work, acquisition of data, analysis and interpretation of data, and drafting the work for intellectual content.

Data availability statement

Data are available upon reasonable request.

REFERENCES

1 Carrasquer P, Torns T, Tejero E, *et al.* El Trabajo Reproductivo. *Pap Rev Sociol* 1998;**55**:95–114. doi:10.5565/rev/papers.1934

2 Benería L. Trabajo productivo/reproductivo, pobreza y políticas de conciliación. *Nómadas* 2006;**24**:8–21.

3 Kan MY, Sullivan O, Gershuny J. Gender convergence in domestic work: Discerning the effects of interactional and institutional barriers from large-scale data. *Sociology* 2011;**45**:234–51. doi:10.1177/0038038510394014

4 Larrañaga, I., Arregui, B., & Arpal J. El trabajo reproductivo o doméstico. *Garceta Sanit* 2004;**18**:31–7.

For peer review only

5 Rohlfs I, Borrell C, Artazcoz L, *et al.* La importancia de la perspectiva de género en las encuestas de salud. *Gac Sanit* 2000;**14**:146–55. doi:10.1016/S0213-9111(00)71448-8

6 de Alba Teniente E. Experience of empowering women through sport. Case study of marathon runners and ultra marathon runners. *Razón* 2017;**21**:281–94.

7 Pérez CB. Feminism, gender and reproductive work. *AusArt J Res Art* 2017;**5**:103–15. doi:10.1387/ausart.17782

8 Benlloch IM. Los efectos de las asimetrías de género en la salud de las mujeres. *Anu Psicol* 2003;**34**:253–66.

9 Mas AJ, Carmona L, Valverde M, *et al.* Prevalence and impact of fibromyalgia on function and quality of life in individuals from the general population: results from a nationwide study in Spain. *Clin Exp Rheumatol* 2008;**26**:519.

10 Estévez-López F, Segura-Jiménez V, Álvarez-Gallardo IC, *et al.* Adaptation profiles comprising objective and subjective measures in fibromyalgia: The al-Ándalus project. *Rheumatol (United Kingdom)* 2017;**56**:2015–24. doi:10.1093/rheumatology/kex302

11 Wolfe F, Smythe HA, Yunus MB, *et al.* The American College of Rheumatology 1990 Criteria for the Classification of Fibromyalgia. Report of the Multicenter Criteria Committee. *Arthritis Rheum* 1990;**33**:160–72. doi:10.1002/art.1780330203

12 Luque-Reca O, Pulido-Martos M, Gavilán-Carrera B, *et al.* Emotional intelligence impairments in women with fibromyalgia: Associations with widespread pain. *J Health Psychol* 2019;:1359105319890916.

13 Palstam A, Bjersing JL, Mannerkorpi K. Which aspects of health differ between working and nonworking women with fibromyalgia? A cross-sectional study of work status and health. *BMC Public Health* 2012;**12**. doi:10.1186/1471-2458-12-1076

14 Reisine S, Fifield J, Walsh S, *et al.* Employment and health status changes among women with fibromyalgia: A five-year study. *Arthritis Care Res* 2008;**59**:1735–41. doi:10.1002/art.24309

For peer review only

15 Segura-Jimenez V, Borges-Cosic M, Soriano-Maldonado A, *et al.* Association of sedentary time and physical activity with pain, fatigue, and impact of fibromyalgia: the al-andalus study. *Scand J Med Sci Sports* 2017;**27**:83–92. doi:10.1111/sms.12630

16 Gavilán-Carrera B, Segura-Jiménez V, Mekary RA, *et al.* Substituting sedentary time with physical activity in fibromyalgia: association with quality of life and impact of the disease. The al-Ándalus project. *Arthritis Care Res (Hoboken)* 2019;**71**:281–9.

17 Ellingson LD, Shields MR, Stegner AJ, *et al.* Physical Activity, Sustained Sedentary Behavior, and Pain Modulation in Women With Fibromyalgia. *J Pain* 2012;**13**:195–206. doi:10.1016/j.jpain.2011.11.001

18 Ministerio de Educación Cultura y Deporte. Encuesta de Hábitos Deportivos en España 2015. 2015.

19 Segura-Jimenez V, Alvarez-Gallardo IC, Carbonell-Baeza A, *et al.* Fibromyalgia has a larger impact on physical health than on psychological health, yet both are markedly affected: The al-Andalus project. *Semin Arthritis Rheum* 2015;**44**:563–70. doi:10.1016/j.semarthrit.2014.09.010

20 Folstein MF, Folstein SE, McHugh PR. “Mini-mental state”: a practical method for grading the cognitive state of patients for the clinician. *J Psychiatr Res* 1975;**12**:189–98.

21 Mannerkorpi K, Hernelid C. Leisure time physical activity instrument and physical activity at home and work instrument. Development, face validity, construct validity and test-retest reliability for subjects with fibromyalgia. *Disabil Rehabil* 2005;**27**:695–701. doi:10.1080/09638280400009063

22 Rosenberg DE, Norman GJ, Wagner N, *et al.* Reliability and validity of the Sedentary Behavior Questionnaire (SBQ) for adults. *J Phys Act Health* 2010;**7**:697–705. doi:10.1123/jpah.7.6.697

23 Munguía-izquierdo D, Pulido-martos M, Acosta FM, *et al.* Objective and subjective measures of physical functioning in women with fibromyalgia : what type of measure is associated most

For peer review only

clearly with subjective well-being ? *Disabil Rehabil* 2019;**0**:1–8. doi:10.1080/09638288.2019.1671503

24 Rivera J, Gonzalez T. The Fibromyalgia Impact Questionnaire: A validated Spanish version to assess the health status in women with fibromyalgia. *Clin Exp Rheumatol* 2004;**22**:554–60.

25 Martínez del Castillo J, Martin Rodriguez M, Hierro Pinés D del, *et al.* Barriers to the physical activity in the adult and alternative women of conciliation. *Act física y Deport Cienc y profesión* 2008;**9**:12–24.

26 Vázquez Gómez B. Mujeres y actividades físico-deportivas (Investigaciones en Ciencias del Deporte, nº35). *Madrid Cons Super Deport* 2002.

27 Bopp M, Child S, Campbell M. Factors Associated with Active Commuting to Work Among Women. *Women Health* 2014;**54**:212–31. doi:10.1080/03630242.2014.883663

28 Vázquez Gómez B. Nuevos Retos para el Deporte y las Mujeres en el Siglo XXI. In: *Actas del Congreso Mujer y Deporte, Bilbao*. 2001.

29 Segura-Jiménez V, Álvarez-Gallardo IC, Estévez-López F, *et al.* Differences in Sedentary Time and Physical Activity Between Female Patients With Fibromyalgia and Healthy Controls: The al-Ándalus Project. *Arthritis Rheumatol* 2015;**67**:3047–57. doi:10.1002/art.39252

30 Spitze G, Loscocco KA. The labor of sisyphus?: women's and men's reactions to housework. *Soc Sci Q* 2000;**81**:1087–101.

31 Briones-Vozmediano E, Ronda-Pérez E, Vives-Cases C. Percepciones de pacientes con fibromialgia sobre el impacto de la enfermedad en el ámbito laboral. *Aten Primaria* 2015;**47**:205–12. doi:10.1016/j.aprim.2014.06.002

32 Estevez-Lopez F. *Fibromyalgia: adapted, positive, and fit people: Breaking stereotypes!* 2018.

33 Gómez Ortíz V. Estrés y Salud en mujeres que desempeñan múltiples roles. *Av en Psicol Latinoam* 2004;**22**:117–28.

For peer review only

34 Mazzei C. Producción y reproducción: la mujer y la división socio-sexual del trabajo. *Rumbos TS, Univ Cent Chile* 2013;**VII**, **N°8**:128–42.

35 Álvarez-gallardo IC, Estévez-lópez F, Torres-aguilar XC, *et al.* Physical activity , sedentary behaviour , physical fitness , and cognitive performance in women with fibromyalgia who engage in reproductive and productive work : the al-Ándalus project. *Clin Rheumatol* 2019;**38**:3585–93.

36 Csizmadi I, Lo Siou G, Friedenreich CM, *et al.* Hours spent and energy expended in physical activity domains: Results from The Tomorrow Project cohort in Alberta, Canada. *Int J Behav Nutr Phys Act* 2011;**8**. doi:10.1186/1479-5868-8-110

37 Dailey DL, Keffala VJ, Sluka KA. Do cognitive and physical fatigue tasks enhance pain, cognitive fatigue, and physical fatigue in people with fibromyalgia? *Arthritis Care Res* 2015;**67**:288–96. doi:10.1002/acr.22417

38 Galiano OD, Sañudo CB. Aplicación del ejercicio físico como terapia en medicina del trabajo para pacientes con fibromialgia. *Med Segur Trab (Madr)* 2013;**59**:310–21. doi:10.4321/S0465-546X2013000300003

39 Cadenas-Sanchez C, Ruiz-Ruiz J. Effect of a physical activity programme in patients with fibromyalgia: A systematic review. *Med Clin (Barc)* 2014;**143**:548–53. doi:10.1016/j.medcli.2013.12.010

40 Bossema ER, Kool MB, Cornet D, *et al.* Characteristics of suitable work from the perspective of patients with fibromyalgia. *Rheumatology* 2012;**51**:311–8. doi:10.1093/rheumatology/ker312

41 Kivimäki M, Leino-Arjas P, Kaila-Kangas L, *et al.* Increased absence due to sickness among employees with fibromyalgia. *Ann Rheum Dis* 2007;**66**:65–9.

42 Henriksson CM, Liedberg GM, Gerdle B. Women with fibromyalgia: work and rehabilitation. *Disabil Rehabil* 2005;**27**:685–94.

For peer review only

43 Segura-Jimenez V, Alvarez-Gallardo IC, Romero-zurita A, *et al.* Comparison of Physical Activity Using Questionnaires (Leisure Time Physical Activity Instrument and Physical Activity at Home and Work Instrument) and Accelerometry in Fibromyalgia Patients : The al-Ándalus Project. *Arch Phys Med Rehabil* 2014;**95**:1903–11. doi:10.1016/j.apmr.2014.05.015

44 Estévez-López F, Martinez-Tellez B, Ruiz JR. Physical fitness and cancer. *Lancet Oncol* 2017;**18**.

45 Kerr J, Anderson C, Lippman SM. Physical activity, sedentary behaviour, diet, and cancer: an update and emerging new evidence. *Lancet Oncol* 2017;**18**:e457--e471.

46 Estevez-Lopez F, Alvarez-Gallardo IC, Segura-Jimenez V, *et al.* The discordance between subjectively and objectively measured physical function in women with fibromyalgia: association with catastrophizing and self-efficacy cognitions. The al-Andalus project. *Disabil Rehabil* 2018;**40**:329–37. doi:10.1080/09638288.2016.1258737

47 Chau JY, van der Ploeg HP, Merom D, *et al.* Cross-sectional associations between occupational and leisure-time sitting, physical activity and obesity in working adults. *Prev Med (Baltim)* 2012;**54**:195–200. doi:10.1016/j.ypmed.2011.12.020

48 Kurita S, Shibata A, Ishii K, *et al.* Patterns of objectively assessed sedentary time and physical activity among Japanese workers: A cross-sectional observational study. *BMJ Open* 2019;**9**:1–9. doi:10.1136/bmjopen-2018-021690

For peer review only

For peer review only

Table 1. Sociodemographic characteristics of the participants (*n*=258)

Educational level		
Incomplete studies	25	(9.7)
Primary school	132	(51.2)
Secondary school	65	(25.2)
University degree	36	(14.0)
Marital status		
Married	201	(77.9)
Single	14	(5.4)
Separated/divorced	26	(10.1)
Widowed	17	(6.6)
Current working status		
Reproductive work	143	(55.4)
Productive work	115	(44.6)
	Mean	(SD)
Age	51.4	(7.9)
Fat percentage (%)	40.2	(7.3)
Total number of tender points	16.6	(2.0)
Leisure-time PA (LTPAI, min/week)		
Light (< 3 METs)	178.9	(143.0)
Moderate (3-6 METs)	62.8	(100.3)
Vigorous (> 6 METs)	16.5	(43.6)
PA at home (PAHWI, min/week)		
Light (< 3 METs)	1188.8	(786.2)
Moderate (3-6 METs)	544.4	(521.0)
Vigorous (> 6 METs)	70.4	(249.2)
PA at work (reproductive and productive, min/week)		
Light	1542.5	(1003.2)
Moderate	607.2	(553.1)
Vigorous	86.9	(258.8)
Total sedentary time (SBQ)		
Monday to Friday (min/weekday)	533.1	(261.2)
During the weekend (min/weekend day)	566.2	(257.6)

For peer review only

SD, standard deviation; PA, physical activity; METs, metabolic index measurement unit; LTPAI, Leisure-Time Physical Activity Instrument; PAHWI, Physical Activity at Home and Work Instrument; SBQ, Sedentary Behavior Questionnaire.

Figure 1. Flow diagram for study participants

Figure 2. Differences in physical activity at home of light intensity between women with fibromyalgia who only performed reproductive work and those who also performed productive work (P<0.001)

Figure 3. Differences in physical activity work (i.e., house + work) of light intensity between women with fibromyalgia who only performed reproductive work and those who also performed productive work (P=0.001)

Figure 4. Differences in total physical activity (i.e., the addition of leisure time, house, and work) of light intensity between women with fibromyalgia who only performed reproductive work and those who also performed productive work (P<0.001)

Figure 5. Differences in total physical activity (i.e., the addition of leisure time, house, and work) of moderate intensity between women with fibromyalgia who only performed reproductive work and those who also performed productive work (P=0.004)

For peer review only

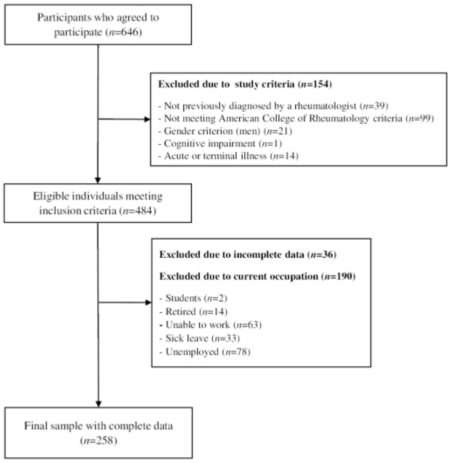

Figure 1. Flow diagram for study participants

338x190mm (200 x 200 DPI)

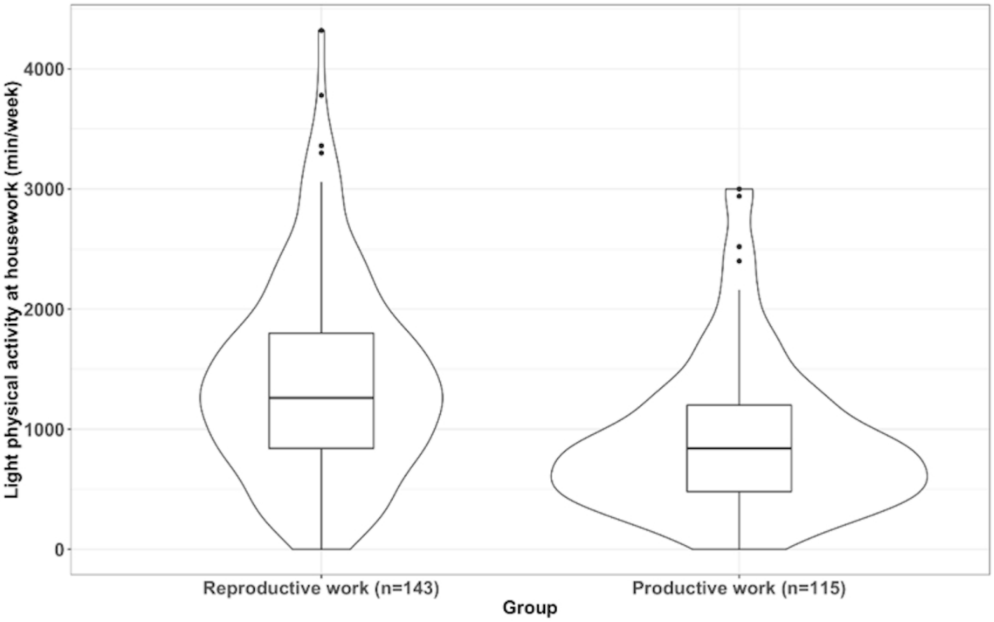

Figure 2. Differences in physical activity at home of light intensity between women with fibromyalgia who only performed reproductive work and those who also performed productive work (P<0.001)

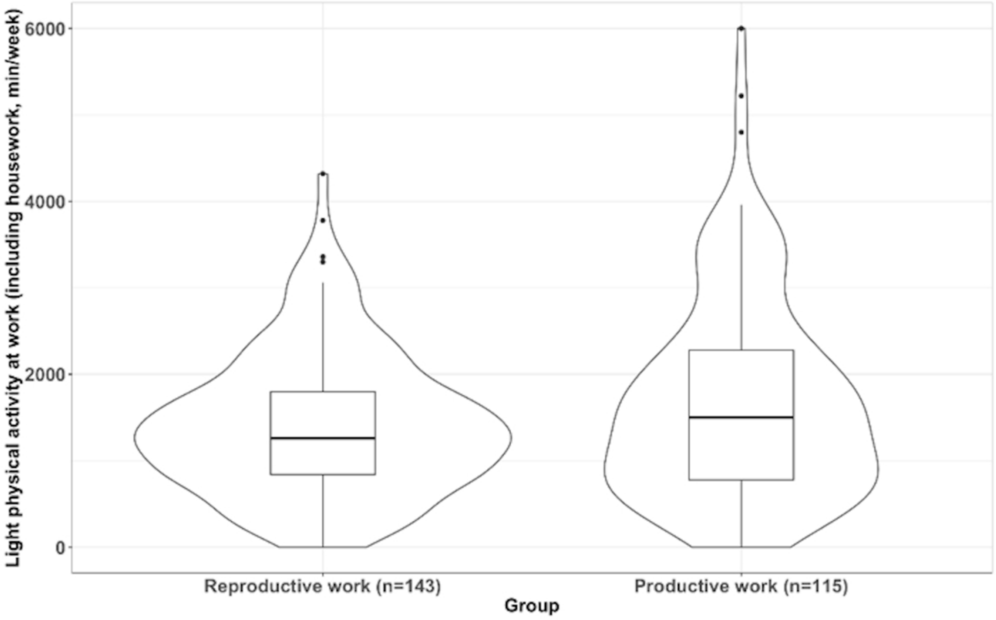

Figure 3. Differences in physical activity work (i.e., house + work) of light intensity between women with fibromyalgia who only performed reproductive work and those who also performed productive work (P=0.001)

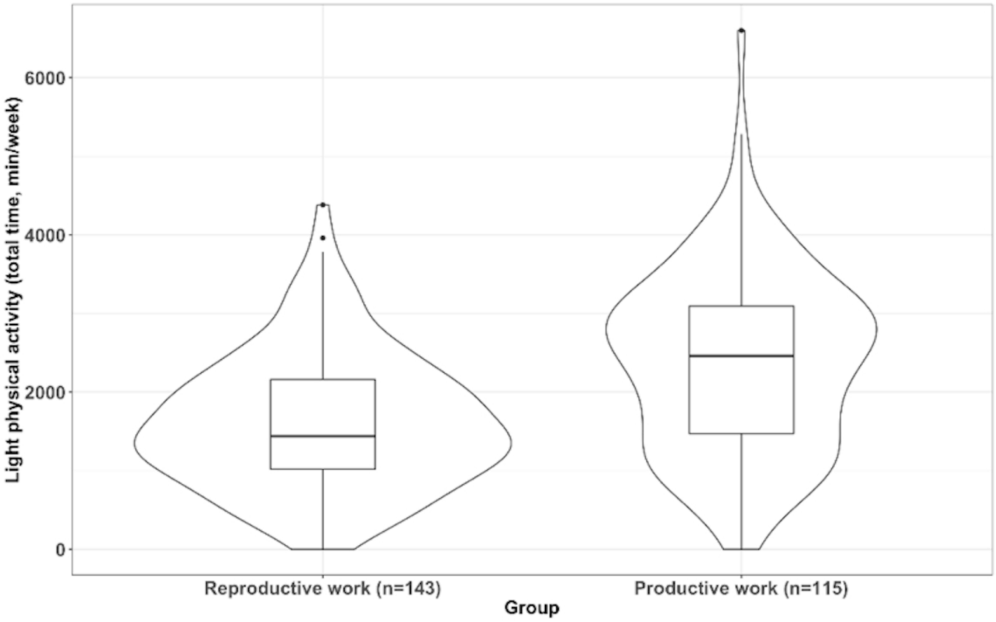

Figure 4. Differences in total physical activity (i.e., the addition of leisure time, house, and work) of light intensity between women with fibromyalgia who only performed reproductive work and those who also performed productive work (P<0.001)

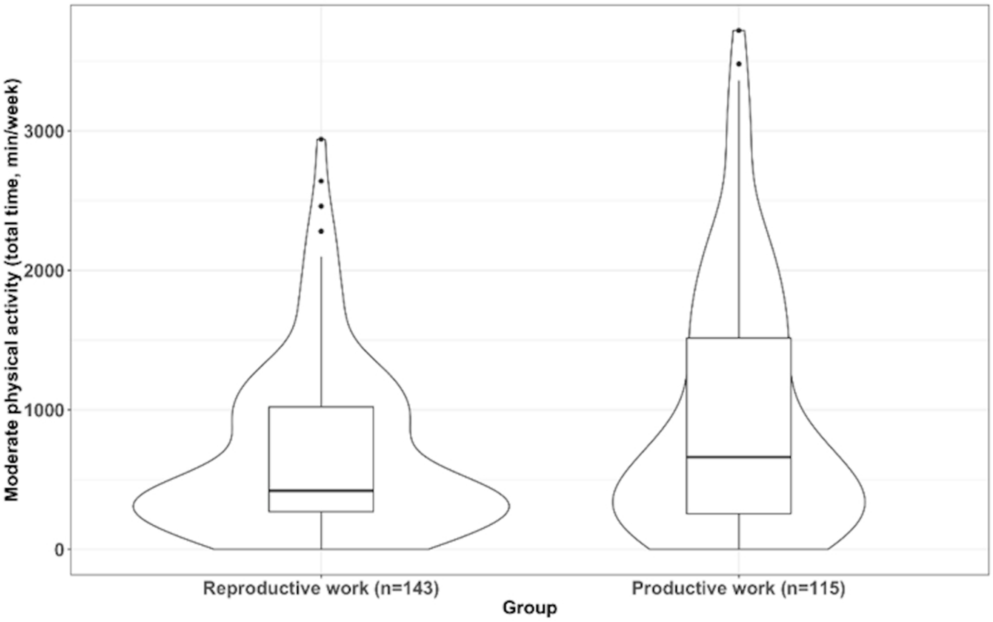

Figure 5. Differences in total physical activity (i.e., the addition of leisure time, house, and work) of moderate intensity between women with fibromyalgia who only performed reproductive work and those who also performed productive work (P=0.004)

STROBE Statement—checklist of items that should be included in reports of observational studies

	Item No.	Recommendation	Page No.	Relevant text from manuscript
**Title and abstract**	1	(*a*) Indicate the study's design with a commonly used term in the title or the abstract	1	*"This cross-sectional study involved 258 women with fibromyalgia…"*
		(*b*) Provide in the abstract an informative and balanced summary of what was done and what was found	1	Methods and results sections from the abstract
**Introduction**				
Background/rationale	2	Explain the scientific background and rationale for the investigation being reported	2-3	Mainly 2nd and 3rd paragraph in the introduction section
Objectives	3	State specific objectives, including any prespecified hypotheses	3	*"Therefore, the aim of the present study was to analyze the association between the type of work (productive vs. reproductive), physical activity intensity levels (in leisure time, home, work, and total), and sedentary behavior in women with fibromyalgia"*
**Methods**				
Study design	4	Present key elements of study design early in the paper	3-4	1st and 2nd paragraph in the methods section
Setting	5	Describe the setting, locations, and relevant dates, including periods of recruitment, exposure, follow-up, and data collection	3-4	1st and 2nd paragraph in the methods section
Participants	6	(*a*) *Cohort study*—Give the eligibility criteria, and the sources and methods of selection of participants. Describe methods of follow-up<br>*Case-control study*—Give the eligibility criteria, and the sources and methods of case ascertainment and control selection. Give the rationale for the choice of cases and controls<br>*Cross-sectional study*—Give the eligibility criteria, and the sources and methods of selection of participants	3	*"The inclusion criteria for the present study were: i) to have been diagnosed with fibromyalgia; ii) to meet the 1990 ACR criteria [10]; iii) not to suffer from any acute or terminal illness, or serious cognitive impairment; iv) to be a woman."*
		(*b*) *Cohort study*—For matched studies, give matching criteria and number of exposed and unexposed<br>*Case-control study*—For matched studies, give matching criteria and the number of controls per case	N/A	

For peer review only - http://bmjopen.bmj.com/site/about/guidelines.xhtml

Variables	7	Clearly define all outcomes, exposures, predictors, potential confounders, and effect modifiers. Give diagnostic criteria, if applicable	4-7	Each paragraph describes diagnostic criteria, outcomes, predictors, and potential confounders.
Data sources/ measurement	8*	For each variable of interest, give sources of data and details of methods of assessment (measurement). Describe comparability of assessment methods if there is more than one group	4-7	Each paragraph describes assessment method of diagnostic criteria, outcomes, predictors, and potential confounders.
Bias	9	Describe any efforts to address potential sources of bias		
Study size	10	Explain how the study size was arrived at	3 and 8	Initial sample size in page 3 *"This study belongs to al-Ándalus project, in which a representative sample of women with fibromyalgia from Andalusia (Spain) was examined [17]."* Final sample size in page 8 "*The flowchart of the participants included in the present study is shown in Figure 1.*"

Quantitative variables	11	Explain how quantitative variables were handled in the analyses. If applicable, describe which groupings were chosen and why	7	Two paragraphs included in the statistical analysis section
Statistical methods	12	(*a*) Describe all statistical methods, including those used to control for confounding	7	Two paragraphs included in the statistical analysis section
		(*b*) Describe any methods used to examine subgroups and interactions	N/A	
		(*c*) Explain how missing data were addressed	Figure 1	
		(*d*) *Cohort study*—If applicable, explain how loss to follow-up was addressed<br>*Case-control study*—If applicable, explain how matching of cases and controls was addressed<br>*Cross-sectional study*—If applicable, describe analytical methods taking account of sampling strategy	3	*"This study belongs to al-Ándalus project, in which a representative sample of women with fibromyalgia from Andalusia (Spain) was examined [17]."*
		(*e*) Describe any sensitivity analyses	8	*"age, fat percentage, education level, and marital status were included as covariates in all the analyses described below. Unadjusted analyses and analyses additionally*

For peer review only - http://bmjopen.bmj.com/site/about/guidelines.xhtml

				accounting for disease severity are included as supplementary material"
**Results**				
Participants	13*	(a) Report numbers of individuals at each stage of study—eg numbers potentially eligible, examined for eligibility, confirmed eligible, included in the study, completing follow-up, and analysed	8	*"The flowchart of the participants included in the present study is shown in Figure 1."*
		(b) Give reasons for non-participation at each stage	8	*"The flowchart of the participants included in the present study is shown in Figure 1."*
		(c) Consider use of a flow diagram	8	*"The flowchart of the participants included in the present study is shown in Figure 1."*
Descriptive data	14*	(a) Give characteristics of study participants (eg demographic, clinical, social) and information on exposures and potential confounders	20	Table 1
		(b) Indicate number of participants with missing data for each variable of interest	8	*"The flowchart of the participants included in the present study is shown in Figure 1."*
		(c) *Cohort study*—Summarise follow-up time (eg, average and total amount)	N/A	
Outcome data	15*	*Cohort study*—Report numbers of outcome events or summary measures over time	N/A	
		Case-control study—Report numbers in each exposure category, or summary measures of exposure	N/A	
		Cross-sectional study—Report numbers of outcome events or summary measures	20	Table 1
Main results	16	(*a*) Give unadjusted estimates and, if applicable, confounder-adjusted estimates and their precision (eg, 95% confidence interval). Make clear which confounders were adjusted for and why they were included	7	Supplementary table 2 and page 7 *"Given that significant differences between groups emerged for all these variables (with the exception of total number of tender points), age, fat percentage, education level, and marital status were included as covariates in all the analyses described below."*
		(*b*) Report category boundaries when continuous variables were categorized	N/A	
		(*c*) If relevant, consider translating estimates of relative risk into absolute risk for a meaningful time period	N/A	
Other analyses	17	Report other analyses done—eg analyses of subgroups and interactions, and sensitivity analyses	7	*"Unadjusted analyses and analyses additionally accounting for disease severity*

For peer review only

				are included as supplementary material (supplementary tables 2 and 3)".
**Discussion**				
Key results	18	Summarise key results with reference to study objectives	9	1st paragraph in the discussion section
Limitations	19	Discuss limitations of the study, taking into account sources of potential bias or imprecision. Discuss both direction and magnitude of any potential bias	12	Limitations paragraph "*The conclusions of the present study must be taken bearing its limitations in mind. A cross-sectional design was used, so the results cannot be deemed as causal…."*
Interpretation	20	Give a cautious overall interpretation of results considering objectives, limitations, multiplicity of analyses, results from similar studies, and other relevant evidence	13	Last paragraph of the discussion *"Given the cross-sectional design of this study, future longitudinal research is warranted in order to address the causality of the present findings. If they are corroborated…"*
Generalisability	21	Discuss the generalisability (external validity) of the study results	13	Last paragraph of the discussion
**Other information**				
Funding	22	Give the source of funding and the role of the funders for the present study and, if applicable, for the original study on which the present article is based	13	Funding paragraph *"This work was supported by the Spanish Ministries of Economy and Competitiveness (I+D+i DEP2010-15639; I+D+i DEP2013-40908-R) …. The funders of this study did not have any role in the study design, data collection and analyses, decision to publish or preparation of the manuscript."*

*Give information separately for cases and controls in case-control studies and, if applicable, for exposed and unexposed groups in cohort and cross-sectional studies.

Note: An Explanation and Elaboration article discusses each checklist item and gives methodological background and published examples of transparent reporting. The STROBE checklist is best used in conjunction with this article (freely available on the Web sites of PLoS Medicine at http://www.plosmedicine.org/, Annals of Internal Medicine at http://www.annals.org/, and Epidemiology at http://www.epidem.com/). Information on the STROBE Initiative is available at www.strobe-statement.org.

For peer review only - http://bmjopen.bmj.com/site/about/guidelines.xhtml

Supplementary Table 2. Mean difference in physical activity variables between women with fibromyalgia with a reproductive work only (n=143) and those with a reproductive + productive work (n=115)

	Light Intensity						Moderate Intensity						Vigorous Intensity					
	Mean difference	95 % CI		P_1	P_2	P_3	Mean difference	95 % CI		P_1	P_2	P_3	Mean difference	95 % CI		P_1	P_2	P_3
Leisure-time PA	4.875	-30.458	40.208	0.786	0.889	0.513	-10.967	-35.706	13.771	0.383	0.355	0.558	-5.980	-16.720	4.760	0.274	0.221	0.268
PA at home	**491.941**	**307.317**	**676.565**	**<0.001**	**<0.001**	**<0.001**	**165.643**	**38.513**	**292.773**	**0.011**	0.081	0.088	6.539	-55.049	68.127	0.835	0.751	0.787
PA at work	**-256.389**	**-502.304**	**-10.473**	**0.041**	**0.001**	**0.002**	**154.676**	**19.318**	**290.033**	**0.025**	0.142	0.135	0.559	-63.411	64.529	0.986	0.920	0.940
Total PA	**-752.616**	**-1000.376**	**-504.857**	**<0.001**	**<0.001**	**<0.001**	**-261.011**	**-445.530**	**-76.492**	**0.006**	**0.004**	**0.006**	-79.799	-166.660	7.061	0.072	0.089	0.079

CI: confidence interval. PA: physical activity. Reproductive + productive work was used as reference category in all comparisons.

Mean difference and P_1 show the results for unadjusted analysis

$P_{2:}$ Analysis adjusted for age, fat percentage, education level, and marital status

P_3: Analysis adjusted for age, fat percentage, education level, marital status, and disease severity

Supplementary Table 3. Mean difference in sedentary behaviour variables between women with fibromyalgia with a reproductive work only (n=143) and those with a reproductive + productive work (n=115)

	Mean difference	95 % CI		P_1	P_2	P_3
Total sedentary Time (Monday to Friday)	-312.514	-632.988	7.959	0.056	0.308	0.207
Total sedentary Time (Weekend)	-63.736	-190.825	63.353	0.324	0.749	0.880

CI: confidence interval. Reproductive + productive work was used as reference category in all comparisons.

Mean difference and P_1 show the results for unadjusted analysis

$P_{2:}$ Analysis adjusted for age, fat percentage, education level, and marital status

P_3: Analysis adjusted for age, fat percentage, education level, marital status, and total disease severity

For peer review only
